# Regulatory B cells contribute to allergen-encapsulating nanoparticle immunotherapy efficacy for food allergy

**DOI:** 10.1172/jci.insight.199988

**Published:** 2026-06-09

**Authors:** Laila M. Rad, Michael N. Saunders, Laura A. Williams, Katarzyna W. Janczak, Chris Dorsett, Kate V. Griffin, Elizabeth J. Bealer, Jeffrey A. Ma, Sayre A. Tillery, Jyotirmoy Roy, Stephen D. Miller, Jessica J. O’Konek, Lonnie D. Shea

**Affiliations:** 1 Department of Biomedical Engineering and; 2 Mary H. Weiser Food Allergy Center, University of Michigan, Ann Arbor, Michigan, USA.; 3 Department of Microbiology-Immunology and; 4 Center for Human Immunology, Northwestern University, Chicago, Illinois, USA.; 5 Department of Chemical Engineering, University of Michigan, Ann Arbor, Michigan, USA.

**Keywords:** Cell biology, Immunology, Allergy, B cells, Nanotechnology

## Abstract

B cells contribute to the pathogenesis of food allergies as they induce allergen-specific antibody production. Clinically used allergen-specific immunotherapies have been shown to induce regulatory B cell subsets as well as target and reduce allergy-driving B cell functions. This report aims to elucidate the contribution of regulatory B cells to an allergen-encapsulating nanoparticle (aeNP) immunotherapy in a murine model of food allergy. In this model, B cells directly associated with aeNPs. CD20^+^ B cell depletion after aeNP treatment increased the number of mice with severe allergic reactions during oral food challenges and reduced the expansion of regulatory immune cells including CD103^+^ DCs and CCR9^+^ gut-homing Tregs, indicating that B cells are a component of aeNP immunomodulation. B cell communication in the gastrointestinal tract of aeNP-treated mice identified CD23 signaling as a potential inducer of regulatory CD103^+^ DC functions and disrupter of allergy-driving B cell–T cell communication. These tolerogenic signaling patterns were also identified in IL-10^+^ B cells, which are known to impart regulatory immune effects in both murine and human disease. Ultimately, B cells are a component of the complex immunomodulation leading to aeNP efficacy at reducing allergic reactivity.

## Abstract 2

An effective preclinical allergen-specific nanoparticle immunotherapy for food allergies induces a multifaceted immune modulation, with contributions from a subset of regulatory B cells.

## Introduction

Food allergies are an undesired immune response to benign food antigens that can lead to potentially life-threatening clinical symptoms. Incidence of severe anaphylactic reactions is increasing globally (1). Allergen-specific immunotherapy aims to modulate the immune response to a specific allergen without affecting the function of the rest of the immune system. Oral immunotherapy (OIT), a type of allergen-specific immunotherapy, is one of the most researched treatments for food allergies and is clinically used for the treatment of peanut allergy (2–6). OIT requires daily dosing for months to years with possible adverse events. Furthermore, OIT does not induce long-lasting desensitization in most patients and requires constant maintenance allergen dosing to retain reductions in allergic reactivity. Although OIT is hypothesized to act on numerous concurrent immune-mediated mechanisms, T cell modulation is a key part of its efficacy (7, 8). Specifically, OIT has been shown to reduce Th2 cells, potentially through exhaustion and anergy, and to increase Tregs that secrete cytokines such as IL-10 and TGF-β (7, 8).

Clinical trials of allergen-specific immunotherapy approaches have recently investigated a potential role for B cell–dependent mechanisms, including induction of regulatory B cell (Breg) phenotypes (9, 10). B cells play complex roles in IgE-mediated food allergies (11–17). They produce allergen-specific antibodies, maintain memory to these allergens, and differentiate into antibody-secreting plasma cells. In addition to these humoral functions, B cells also have antigen-presenting capabilities that facilitate T cell communication. For OIT specifically, 1 Breg subset is classified by its regulatory humoral effect (18). Patients receiving OIT tend to have higher serum allergen-specific antibody levels and allergen-specific circulating B cells that are isotype-switched, enhancing the ratio of allergen-specific IgG4 to IgE (9, 10, 18, 19). Allergen-specific IgG4 found in patients receiving OIT is thought to act as a neutralizing antibody by preventing IgE-allergen complexes from binding with allergy effector cells, namely mast cells and basophils (9, 10, 19–21). In a clinical trial of cow’s milk allergy OIT, patients who were classified as being in remission versus desensitization had less B cell activation and more Breg markers associated with tolerance on allergen-specific B cells. These clinical results suggest allergen-specific Bregs may be contributing to reducing allergic reactivity in OIT (9). However, OIT results in an incomplete modulation of the B cell response because while allergen-specific IgE levels decrease during OIT, levels remain elevated in the blood. Other immune-targeted therapies have shown efficacy by interfering with pathogenic B cell responses; specifically, clinical trials of allergen-agnostic biologic treatments targeting cytokines and allergen-specific antibodies such as anti-IgE (e.g., omalizumab) and anti-IL4ra (e.g., dupilumab). Although these methods avoid treatment-induced allergen-specific anaphylaxis, they have shown variable therapeutic effects, with the duration of efficacy likely transient and potentially requiring life-long dosing to maintain benefits (22, 23).

Approaches to improve the safety and efficacy of allergen-specific immunotherapy are currently in development and include changing the route of administration (e.g., epicutaneous, sublingual, and subcutaneous) and incorporating adjuvants (e.g., biologics and probiotics) (23). Allergen-encapsulating nanoparticles (aeNPs) delivered i.v. are an allergen-specific immunotherapy that have shown preclinical efficacy at reducing allergic symptoms in murine models of food allergy with only 2 doses (24, 25). The design of aeNPs to have a negative surface charge and 500 nm size targets immune cells in a similar manner as apoptotic cell debris, resulting in preferential aeNP-association with antigen-presenting cell (APC) scavenger receptors, such as MARCO on macrophages (26, 27). The efficacy of this minimal dosing strategy has been shown to be due to aeNPs being phagocytosed by APCs, which induce tolerogenic processes (26–31), such as antigen presentation to T cells to induce regulatory functions (31, 32). Specifically, aeNPs have been shown to reprogram allergen-specific T cells from Th2 phenotypes to regulatory and anergic phenotypes as well as to induce and associate with CD103^+^ DCs, known to induce Treg subtypes (25).

Yet, similar to OIT, these T cell–dependent mechanisms are part of a complex modulation of the immune system. Thus, this report expands on these previous findings by investigating the contribution of B cells as another potential mechanism leading to reduction of allergic responses in a murine model of OVA allergy. Notably, OVA-encapsulating nanoparticle (OVA NP) biodistribution demonstrated a majority of aeNP^+^ cells were B cells. Yet, OVA-specific IgE antibody levels were not reduced by aeNP treatment, and OVA-specific IgG isotypes remained unchanged, suggesting aeNPs do not work by an isotype-switching mechanism. Thus, we investigated other potential B cell communication functions as a mechanism of action. Preventing this B cell–aeNP association using a CD20^+^ B cell–depleting antibody, anti-CD20, identified B cells as a contributor to the induction of regulatory cell types, specifically, to splenic CD103^+^ DC expansion and gut-homing marker expression on small intestine lamina propria (SILP) Tregs. These indications of Breg mechanisms contributing to aeNP efficacy were further supported by splenic and SILP B cells expressing Breg markers after treatment. Single-cell RNA-Seq of OVA NP–treated and allergic control SILP also identified tolerogenic Breg cell crosstalk with APCs and disruption of allergy-driving immune signaling. Flow cytometry and RNA-Seq corroborated the presence of a regulatory CD23^+^ IL-10–secreting B cell subset. Collectively, these studies investigate aeNP treatment for food allergies and their impact on B cell phenotype and communication.

## Results

### A majority of aeNP-associated cells were B cells across major immune organs.

**Figure 1:**
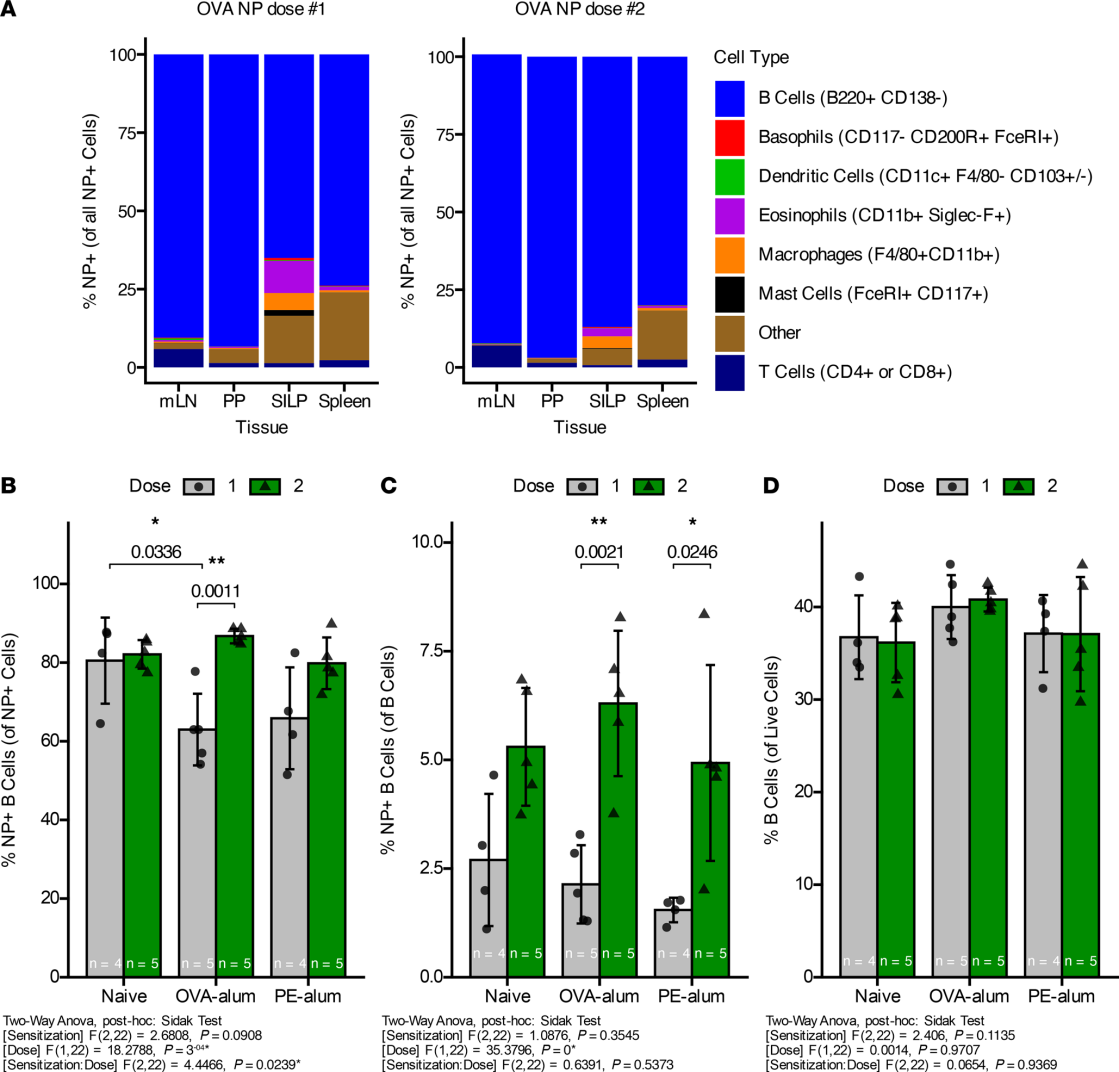
The majority of aeNP^+^ cells are B cells. On days 0 and 7, BALB/cJ mice were sensitized i.p. with either OVA and alum, peanut extract (PE) and alum, or PBS for healthy controls. All mice received treatment with 2.5 mg of i.v. Cy5.5-conjugated OVA NPs on days 14 and 21. Mesenteric lymph nodes (mLNs), Peyer’s patches (PPs), small intestine lamina propria (SILP), and splenocytes were isolated and analyzed via flow cytometry on days 15 and 22 to identify OVA NP–associated cells. (**A**) Biodistribution of OVA NPs with lymphocytes and myeloid cells across tissues in OVA-alum–sensitized mice. (**B**) In the SILP, the percentage of NP^+^ B cells of all NP^+^ cells across different sensitizations. (**C**) In the SILP, the proportion of NP^+^ B cells of all B cells across different sensitizations. (**D**) In the SILP, the percentage of B cells of all live cells across sensitizations. Data shown as mean ± SD; statistically significant differences were identified using 2 way-ANOVA with a post hoc Šidák’s test; *P* values are shown above significance brackets and sample size at the bottom of each bar (*n* = 4–5). *P < 0.05, **P < 0.01.

Initial studies analyzed the biodistribution of aeNPs, specifically characterizing association of OVA NPs with immune cells. Cy5.5-tagged OVA NPs were used to identify cells associating with NPs via flow cytometry 24 hours after i.v. dosing to mice sensitized with OVA plus alum, peanut extract (PE) plus alum, or control PBS (all i.p. delivered). Murine pan–B cell marker B220 identified that over 50% of NP^+^ cells were B cells across all samples analyzed, regardless of tissue — mesenteric lymph nodes, Peyer’s patches, SILP, and spleen — and dose (Figure 1A). In the SILP, after the first dose of OVA NPs, OVA-alum–sensitized mice had a smaller proportion of NP^+^ B cells than nonsensitized controls (Figure 1B). Only in OVA-alum–sensitized mice did the proportion of NP^+^ B cells of all NP^+^ cells increase from the first to second dose (Figure 1B). The proportion of SILP B cells that were NP^+^ increased from the first to second dose only in food allergic conditions — SILP sensitized to OVA-alum and PE-alum (Figure 1C). These changes in NP^+^ B cell proportions were not associated with a change in the proportion of B cells in the SILP (Figure 1D).

### aeNP mechanism of action has a B cell component.

**Figure 2:**
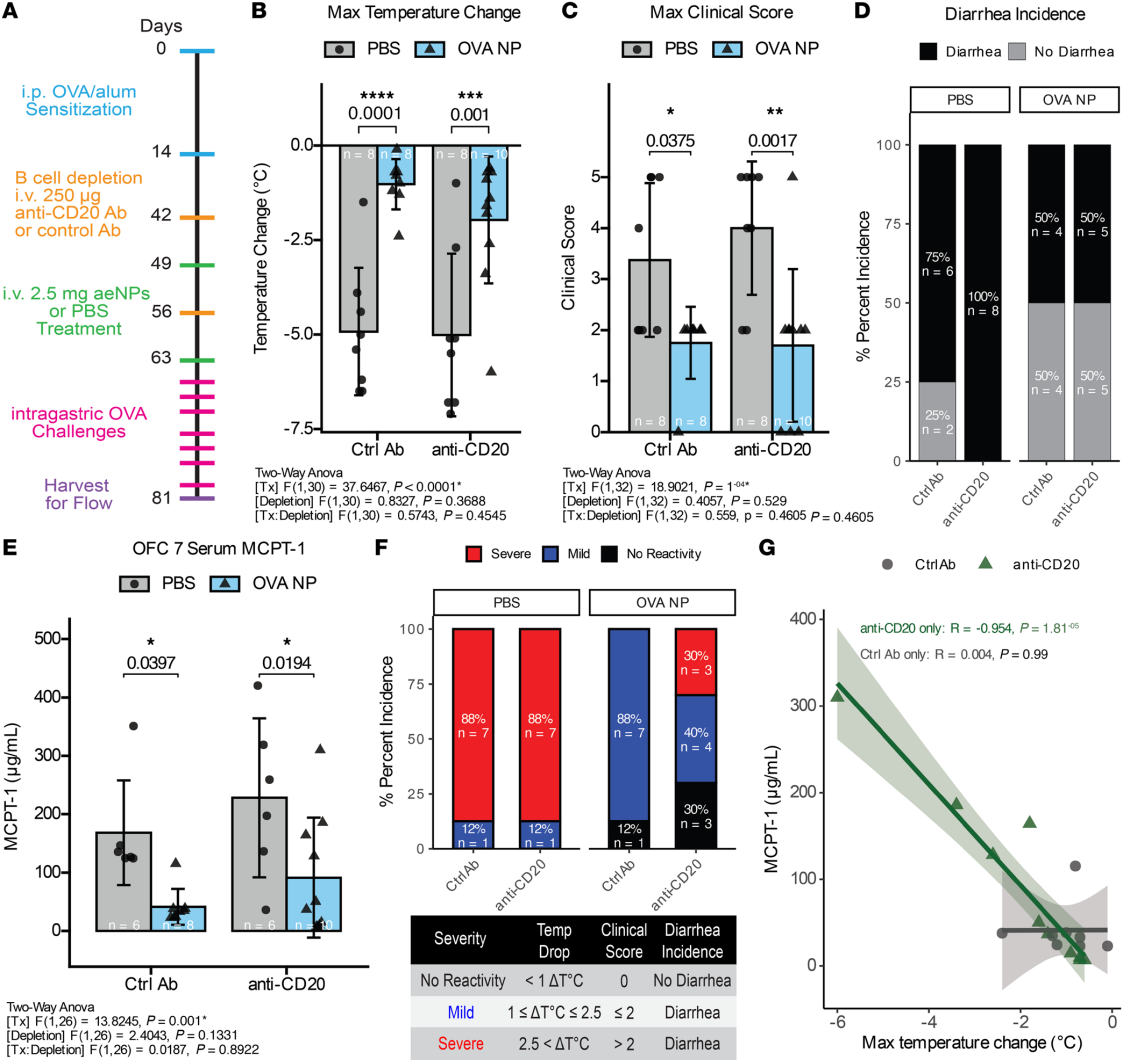
B cell depletion effects on aeNP clinical outcomes. (**A**) B cells were depleted using an anti-CD20 antibody 1 week before each OVA NP dose as shown in the study timeline. Clinical outcomes were assessed by the maximum temperature change between OFC 6 and OFC 7 (**B**), the maximum clinical score between OFC 6 and OFC 7 (**C**), diarrhea incidence (**D**), and serum MCPT-1 levels (**E**). Data shown as mean ± SD; statistically significant differences were identified using 2-way ANOVA with a post hoc Šidák’s test; *P* values are shown above significance brackets and sample size at the bottom of each bar (*n* = 6–10). (**F**) Severity of allergic reactivity was categorized into severe, mild, or no reactivity based on clinical outcomes with the counts and proportions plotted**.** (**G**) Scatter plot of serum MCPT-1 levels and maximum body temperature drops for OVA NP–treated mice with anti-CD20 (*n* = 10) and control antibody (*n* = 8) with linear regression line and 95% CI plotted. Pearson’s correlation coefficients and *P* values of the test of no correlation using Pearson’s product moment correlation are shown on the graph. *P < 0.05, **P < 0.01, ***P < 0.001, ****P < 0.0001.

We next investigated the role of B cells in aeNP-induced tolerance through CD20^+^ B cell depletion during aeNP treatment. OVA-alum–sensitized mice were given 2 doses of OVA NPs 2 weeks apart. Anti-CD20 or control antibody were given as i.v. doses 1 week before each aeNP dose to ensure mature B cells were depleted during the time of treatment (Figure 2A). Mature B cells express CD20 but lose this expression as they differentiate into plasma cells (33). OVA-specific IgG and IgE antibody levels for the control allergic mice (Supplemental Figure 6; supplemental material available online with this article; https://doi.org/10.1172/jci.insight.199988DS1) did not change with anti-CD20 administration. Further, we confirmed CD20^+^ B cell depletion was complete across all tissues of interest (Supplemental Figure 3 and Supplemental Figure 4), and no OVA NP^+^ B cells were present in the anti-CD20–treated mice (Supplemental Figure 4A). However, a small population of B cells was observed in the SILP that were aeNP^+^B220^+^CD138^–^CD20^–^ (Figure 4A). Nevertheless, the biodistribution of OVA NPs did not significantly change in other myeloid immune cell types with anti-CD20 administration (Supplemental Figure 4B).

Allergic reaction severity after oral food challenges (OFCs) 6 and 7 was assessed for aeNP-treated mice with and without CD20^+^ B cell depletion. Comparing across OFC-induced hypothermia, clinical scores, diarrhea incidence, and mast cell protease-1 (MCPT-1) levels (Figure 2, B–E), no significant difference was observed between aeNP-treated mice with and without CD20^+^ B cell depletion. Because these allergic reactivity measures (Figure 2, B–E) had heterogeneity, we stratified the mice into severe reactors, mild reactors, and nonreactors (Figure 2F). OVA NP treatment in the control group (no depletion) was efficacious, as indicated by the absence of severe reactors (0 of 8) and increase in mild and no reactivity groups (8 of 8) relative to the PBS control (7 of 8 severe). The number of mice that were severely reactive with aeNP treatment for the CD20^+^ B cell–depleted mice increased to 3 of 10 (Figure 2F). A strong negative correlation with MCPT-1 levels and body temperature was observed for the aeNP-treated and CD20^+^ B cell–depleted group, yet not observed with aeNP treatment and the control antibody (Figure 2G), which suggests that B cells may have a role in aeNP treatment efficacy by limiting reaction severity.

**Figure 3:**
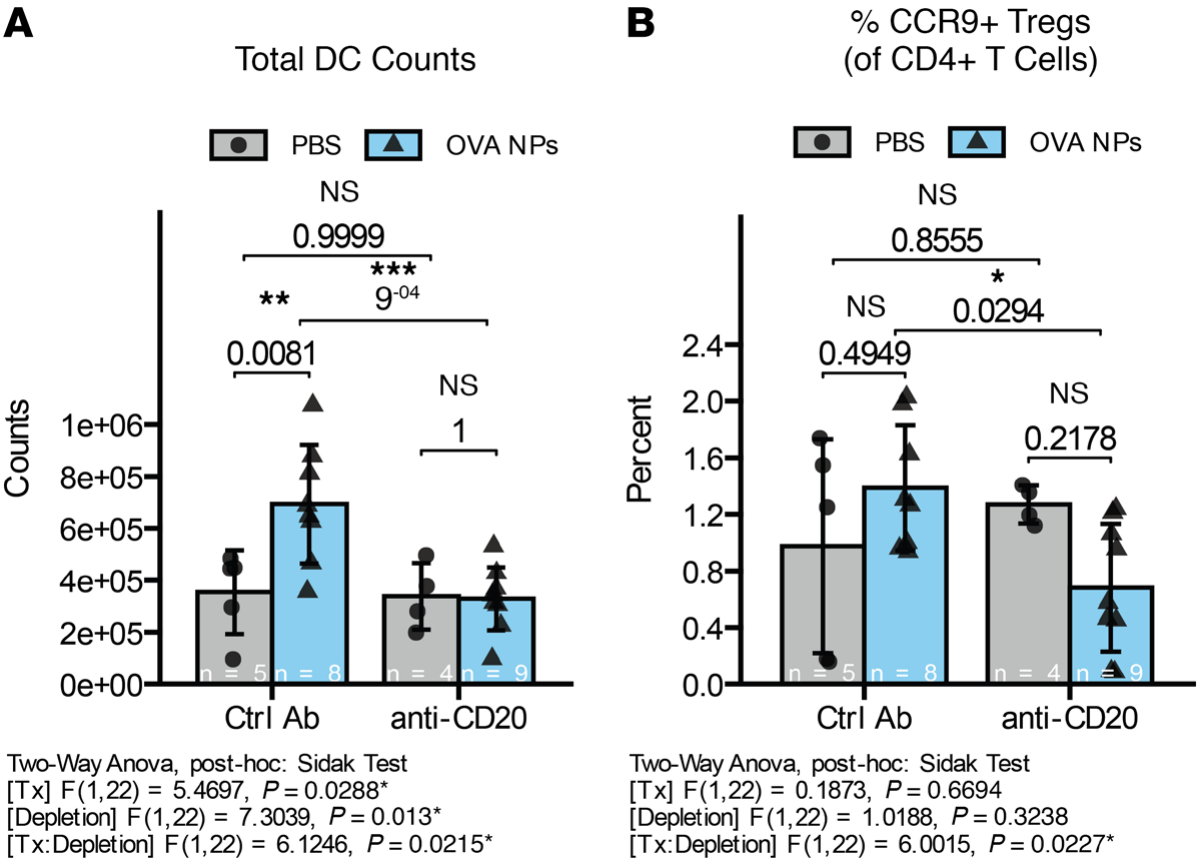
Regulatory cell types induced by aeNP treatment are affected by B cell depletion. One day after OFC 7, spleens and SILP were harvested for flow cytometry to characterize splenic DCs (**A**) and SILP T cells (**B**). (**A**) Splenic CD103^+^ DC counts in OVA NP–treated mice and allergic controls (PBS) with and without B cell depletion. (**B**) The proportion of Tregs expressing the gut-homing marker CCR9 of all CD4^+^ T cells in the SILP in OVA NP–treated mice and allergic controls (PBS) with and without B cell depletion. Data shown as mean ± SD; statistically significant differences were identified using 2-way ANOVA with a post hoc Šidák’s test; adjusted *P* values are shown above significance brackets and sample size at the bottom of each bar (*n* = 4–9). *P < 0.05, **P < 0.01, ***P < 0.001, NS = not significant.

The regulatory mechanisms by which B cells may modulate allergic reactivity were next investigated by comparing cellular responses after OFC with and without CD20^+^ B cell depletion. Because OVA-specific IgE antibody levels were not reduced by aeNP treatment and OVA-specific IgG isotypes remained unchanged (Supplemental Figure 6), we do not suspect aeNP efficacy depends on isotype-switching in B cells. We have previously identified the importance of OVA NP–induced tolerogenic processes occurring in DCs of the spleen and T cells of the SILP (25). Thus, flow cytometry of splenic DCs and SILP T cells was performed after OFC 7 after OVA NP treatment with anti-CD20 B cell depletion or control antibody administration (Figure 2A). In the control antibody group, OVA NP treatment increased splenic F4/80^–^CD11c^+^CD103^+^ DCs from allergic mice (Figure 3A). The CD20^+^ B cell–depleted group did not demonstrate increases in splenic CD103^+^ DCs, suggesting CD20^+^ B cells contribute to splenic CD103^+^ DC expansion during OVA NP treatment. Because CD103^+^ DCs are known to induce gut-homing markers on Tregs (34, 35), we investigated Treg frequency and CCR9 gut-homing marker expression in the SILP. CD20^+^ B cell depletion decreased CCR9-expressing Tregs in OVA NP–treated mice (Figure 3B), suggesting CD20^+^ B cells contribute to expression of gut-homing markers on Tregs. Collectively, CD20^+^ B cell depletion inhibited NP-induced tolerogenic CD103^+^ DC expansion and CCR9 expression on Tregs, indicating a role for CD20^+^ B cells in these regulatory pathways.

### Systemic changes in B cells after OVA NP treatment included an increase in Breg marker expression and suppression of immune activation.

**Figure 4:**
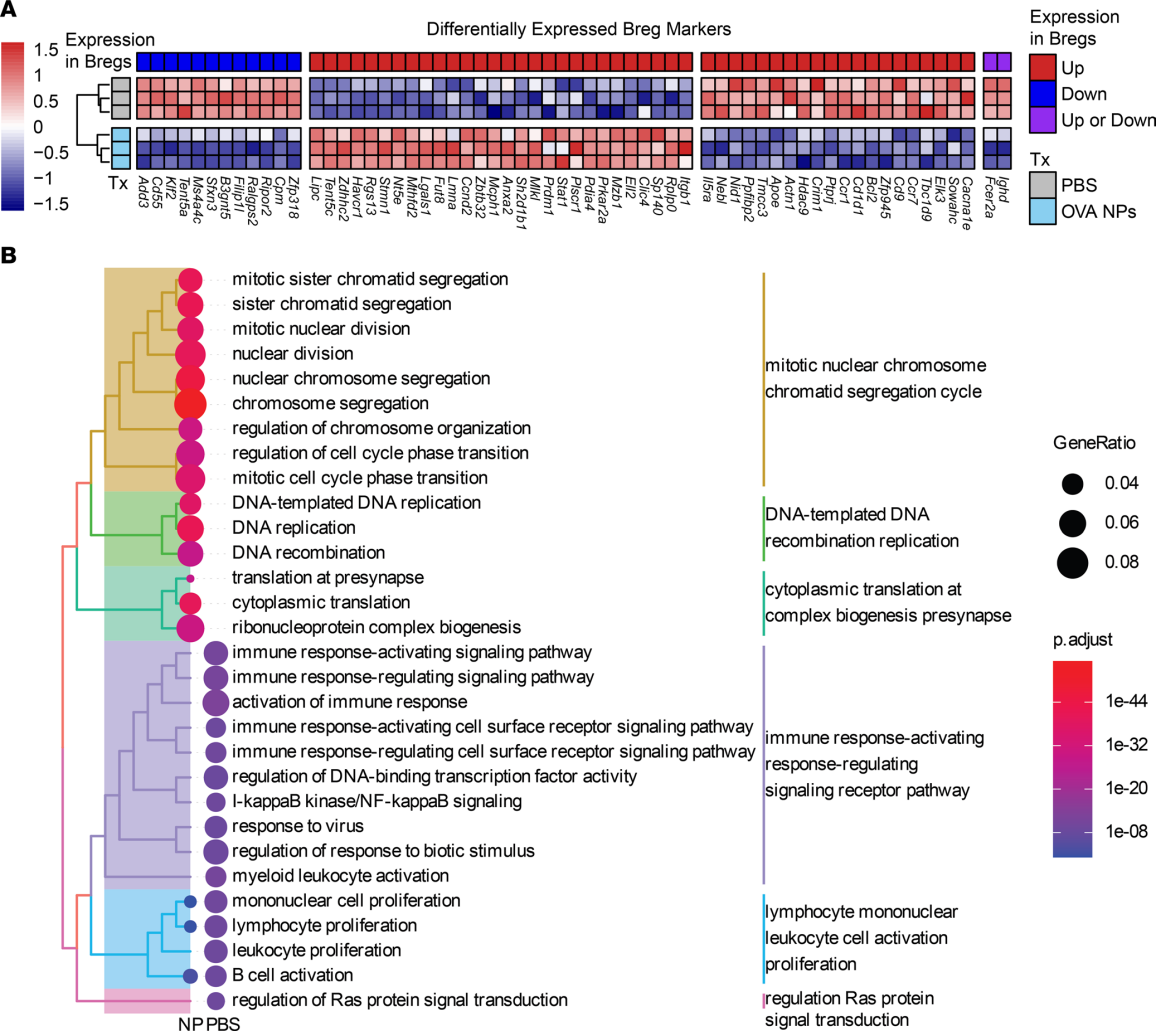
Bulk RNA-Seq of splenic B cells after OVA NP treatment. Mice sensitized with OVA-alum on days 0 and 14 were treated with OVA NPs on days 28 and 42. On day 44, spleens were processed into single-cell suspensions, and B cells were isolated by magnetic-activated cell sorting of negatively selected CD19^+^ cells for bulk RNA-Seq. (**A**) Differentially expressed Breg markers between OVA NP– and PBS-treated mice are displayed in a heatmap of regularized log-transformed data that is centered and scaled across each gene. (**B**) Gene ontology overrepresentation analysis of biological process terms. The data are presented in a tree plot stratified by hierarchical clustering showing the top 15 terms by adjusted *P* value per condition. Five clusters were identified and labeled with a distinct color and the high-frequency words from terms in that cluster. OVA NP–treated mice: *n* = 3; PBS-treated mice: *n* = 3.

Splenic B cells were isolated from mice treated with 2 doses of OVA NPs or PBS controls for bulk RNA-Seq. Because there is not a widely agreed upon set of markers for Bregs, Breg markers were identified from the literature (11, 36, 37) as differentially expressed genes (DEGs) compared with other B cell subsets. These markers include upregulated and downregulated genes from well-defined Breg subsets such as IL-10–secreting B10 cells (11, 36) and from more recently identified Breg markers by comparing Breg subtypes across different organs and with non-Breg B cells (36, 37). Of the 2,024 DEGs between aeNP-treated and allergic mice, 42 genes were associated with Bregs in OVA NP–treated mice and 22 genes in PBS-treated mice. Hierarchical clustering of these genes showed distinct stratification of OVA NP–treated mice from PBS controls (Figure 4A). Breg markers identified in OVA NP–treated mice, which included *Lipc*, *Zbtb32*, *Zdhhc2*, *Rgs13*, *Mlkl*, *Stat1*, *Nt5e*, and *Sh2d1b1*, are associated with a subset of IL-10–secreting B cells, B10 Bregs (36). OVA NP–treated mice also had upregulated expression of several Breg genes common across liver, spleen, bone marrow, peritoneal cavity, and mesenteric lymph node tissue, which included *Ccnd2*, *Sp140*, *Rplp0*, *Pdia4*, and *Mzb1* (36). Additionally, Breg markers identified as upregulated in Bregs compared with non-Breg B cells from a meta-analysis of mouse gene expression datasets were upregulated with OVA NP treatment and included *Clic4*, *Ell2*, *Fut8*, *Itgb1*, *Mcph1*, *Mthfd2*, *Lgals1*, *Anxa2*, *Plscr1*, *Prdm1*, *Prkar2a*, and *Tent5c* (37). Of note, *Itgb1* encodes for the surface receptor CD29, implicated in lymphocyte migration (38). Notably, several B10 Breg markers were also upregulated in allergic controls, including *Cd9*, *Apoe*, *Tbc1d9*, *Nid1*, *Nebl*, *Actn1*, *Zfp945*, *Hdac9*, *Elk3*, *Crim1*, *Ptprj*, and *Ccr1*. An analysis of gene ontology (GO) terms that were overrepresented with the OVA NP treatment confirmed dampening of immune activation pathways (Figure 4B). Hierarchical clustering of enriched GO terms identified 5 distinct clusters that stratified OVA NP–treated and PBS-treated conditions. The PBS condition had enriched terms related to immune activation, whereas the OVA NP condition lacked these immune-activating processes and showed enrichment of cell cycle and protein translation processes. Taken together, this analysis of spleen B cells by bulk RNA-Seq identified changes in gene expression associated with Bregs after OVA NP treatment, which motivated a deeper investigation of B cell communication using single-cell RNA-Seq.

### B cell communication in the SILP of aeNP-treated mice indicates Breg functions.

**Figure 5:**
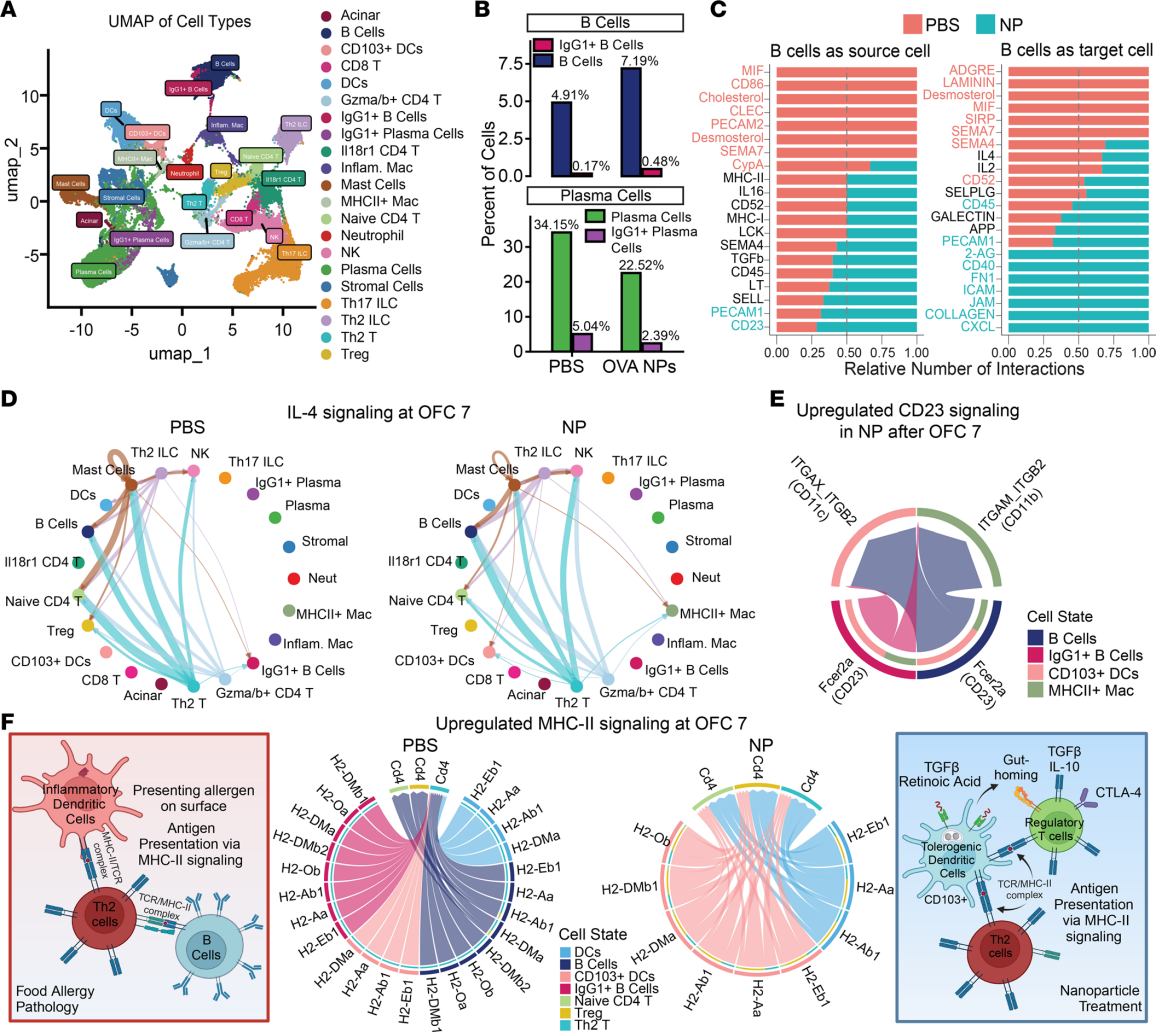
Single-cell RNA-Seq of the SILP identifies changes in B cell communication after aeNP treatment. Mice sensitized with OVA-alum on days 0 and 14 were treated with OVA NPs on days 28 and 42, and SILP were harvested for single-cell RNA-Seq after OFC 7 on day 60. (**A**) Cell types from all samples are shown in the UMAP. (**B**) The proportion of B cell lineage cell types of all cell types by treatment. (**C**) CellChat was used to do receptor-ligand cell communication analysis. Signaling networks at OFC 7 of allergic and OVA NP–treated SILP ranked based on the number of interactions for B cells as source and target cells. Signaling pathway names labeled in red or blue in PBS- or OVA NP–treated mice, respectively, are significantly different by paired Wilcoxon’s test (*P* < 0.05). Circle plot (**D**) or chord diagrams (**E** and **F**) show arrows going from source cells to target cells. Receptor, ligand genes, or cell type names are placed on the outside circumference. (**D**) IL-4 signaling at OFC 7 for both PBS- and OVA NP–treated SILP. (**E**) Upregulated CD23 signaling in SILP of OVA NP–treated mice from PBS controls at OFC 7. (**F**) Upregulated MHC-II signaling at OFC 7 in SILP of OVA NP–treated and PBS-treated mice. Data subsets: only Th2 T cells, Tregs, and naive CD4^+^ T cells were target cells and only DCs, CD103^+^ DCs, B cells, and IgG1^+^ B cells were sender cells. Illustration of immune cell communication was created in BioRender.

We next investigated B cell crosstalk with other immune cells in aeNP-treated SILP using receptor-ligand analysis with CellChat (39). SILP from PBS and aeNP-treated mice were isolated after OFC 7 for single-cell RNA-Seq. We identified 21 cell types comprising immune and stromal cells (Figure 5A). Cell types of the B cell lineage changed in proportion compared with all cell types identified with aeNP therapy (Figure 5B). Plasma cell proportions were greater in allergic mice than in OVA NP–treated mice, whereas B cell proportions were greater in aeNP-treated mice. B cells were involved as source and target cells in numerous immune and metabolic pathways in both allergic and OVA NP–treated mice (Figure 5C). With B cells as the source cell, communication through PECAM-1 signaling was only present in OVA NP–treated SILP. PECAM-1 signaling is thought to prevent B cell activation and maintain immune tolerance (40–43). Within allergic disease, IL-4 is a major Th2 cytokine associated with sensitization. IL-4 signaling was observed in both allergic and OVA NP–treated mice. The number of IL-4 interactions between cell types was similar between groups (Figure 5C), though the specific cell-cell signaling differed (Figure 5D). Allergic mice had IL-4 signaling between Th2 T cells and IgG1^+^ B cells, yet this interaction was not predicted by the data to be present in OVA NP–treated mice (Figure 5D). The OVA NP–treated mice had unique IL-4 interactions associated with Th2 T cells communicating with CD103^+^ DCs and MHC-II^hi^ macrophages.

We next investigated CD23, a low-affinity IgE receptor. This communication pattern was one of the few that were upregulated in the OVA NP–treated SILP B cells relative to the control allergic SILP (Figure 5C). Cell-type biodistribution of OVA NPs showed that association of OVA NPs to B cells was mostly through CD23^+^ B cells (Supplemental Figure 5). Because CD23 is thought to both drive (44) and regulate (45–48) allergic reactivity, the exact mechanisms of CD23 in food allergy immunotherapy (e.g., OIT, sublingual immunotherapy [SLIT]) have not been fully derived. In the SILP, OVA NP–treated mice had upregulated CD23 signaling compared with allergic controls between the CD23 receptor (*Fcer2a*) on B cells and IgG1^+^ B cells with the CD11c receptor (ITGAX_ITGB2) on CD103^+^ DCs and the CD11b receptor (ITGAM_ITGB2) on macrophages (Figure 5E). Since CD23 signaling can lead to allergy-driving B cell–T cell communication, we next investigated MHC-II signaling between B cells, T cells, and DCs (Figure 5F). Allergic mice had upregulated B cell–T cell communication via MHC-II in the SILP, whereas OVA NP–treated mice had upregulated DC–T cell communication; specifically, upregulation of CD103^+^ DC communication to naive CD4^+^ T cells, Th2 T cells, and Tregs. Collectively, the data indicated that OVA NP treatment alters the communication patterns between B cells, T cells, and DCs, with the patterns consistent with regulatory signals that would attenuate allergic responses.

**Figure 6:**
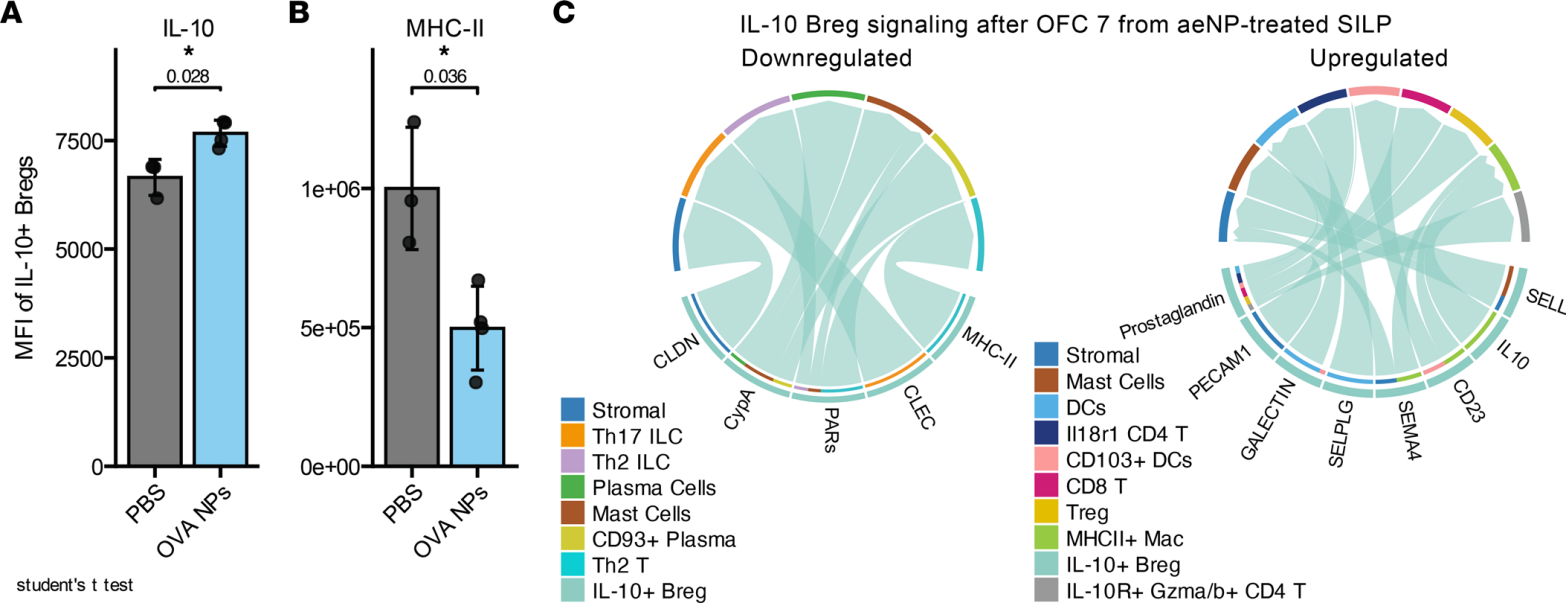
OVA NP treatment induces distinct IL-10^+^ regulatory B cell phenotype and crosstalk. Mice sensitized with OVA-alum on days 0 and 14 were treated with OVA NPs on days 28 and 42, and SILP were harvested for flow cytometry after OFC 7 on day 60. MFI of IL-10 (**A**) and MHC-II (**B**) in IL-10^+^ B cells in allergic (PBS) and OVA NP–treated mice. Data shown as mean ± SD; statistically significant differences were identified using a 2-sided Student’s *t* test; *P* values are shown above significance brackets (PBS: *n* = 3; OVA NP: *n* = 4). (**C**) CellChat was used to do receptor-ligand cell communication analysis of SILP single-cell RNA-Seq data. Chord diagrams of downregulated and upregulated signaling in OVA NP–treated SILP IL-10^+^ B cells as the source cell at OFC 7. *P < 0.05.

B cell immunophenotyping identified an IL-10^+^ Breg phenotype that upregulates CD23 signaling (Figure 6). Consistent with receptor-ligand analysis, gene set enrichment analysis of B cells identified enrichment of regulatory immune gene sets in OVA NP–treated B cells in the SILP compared with allergic controls (Supplemental Figure 13A), which included IL-10 signaling. This indicated the potential for identifying a B cell subset that enacts regulatory functions via IL-10. Immunophenotyping by flow cytometry and single-cell RNA-Seq corroborated the presence of an IL-10–secreting Breg population (Figure 6 and Supplemental Figure 13, B and C). With OVA NP treatment, there was more IL-10 and less MHC-II present compared with allergic controls (Figure 6, A and B). These findings are also analogous to receptor-ligand analysis done on the IL-10^+^ Breg population (Figure 6C). IL-10 and CD23 communication was upregulated by IL-10^+^ Bregs in the SILP of OVA NP–treated mice, showing that IL-10^+^ Bregs communicate via CD23 through engagement with CD11c and CD11b on CD103^+^ DCs and macrophages, respectively (Supplemental Figure 13D). Similarly, MHC-II and proteinase-activated receptors (PARs), along with cyclophilin A (CypA) communication, were downregulated with OVA NP treatment, indicating a reduction in allergy-driving immune cell activation.

## Discussion

In food allergic disease, B cells can produce allergen-specific antibodies, maintain memory to these allergens, and present antigen to T cells that drive allergic responses (11–17). Emerging allergen-specific therapies, such as OIT for food allergies, have shown effective dampening of pathogenic B cell responses by induction of B cell isotype-switching and secretion of allergen-IgE complex–neutralizing antibodies, such as allergen-specific IgG4 (9, 10, 19), as well as through induction of Breg subtypes secreting IL-10 and TGF-β (9). Similarly, allergen-agnostic approaches, including anti-IgE therapies (i.e., omalizumab), indirectly target allergen-specific B cell–mediated immunity by blocking IgE (22, 23). Yet, the effects of these treatments only induce transient desensitization, most likely in part due to a lack of ablation of food allergy–driving allergen-specific memory B cells and plasma cells. Herein, we investigated aeNPs that primarily associate with B cells and serve as an effective treatment of food allergies. We have previously reported aeNPs are effective at reducing allergic responses in food-allergic mice (24, 25) via their effects on CD103^+^ DCs and Treg induction (25). This report expanded on these previous findings by investigating the contribution of B cells to this reduction of allergic responses.

Interestingly, B cell depletion during aeNP treatment did not lead to statistically significant differences in clinical outcomes characterized by anaphylaxis-induced hypothermia, behavioral scoring, diarrhea incidence, and serum MCPT-1 levels. However, the strong negative correlation between serum MCPT-1 levels and body temperature in the CD20^+^ B cell–depleted OVA NP–treated group and not the control OVA NP–treated group may indicate aeNP association with CD20^+^ B cells has a greater role for resolving severe allergies but less so in mild allergies, suggesting complex roles for B cells in aeNP immunotherapy. Paradoxical roles of multiple B cell subsets are reported across different immune pathologies (49) including autoimmunity (50–52) and transplant rejection (53). Analogously, the clinical effects after B cell depletion have been variable depending on the timing of anti-CD20 administration due to different B cell subsets having opposing roles in regulating and driving pathogenesis (49, 50, 53, 54). In experimental autoimmune encephalomyelitis, Bregs have an important role in regulating the severity of disease onset but less of a role during disease progression (50), illustrating that the timing of B cell depletion is important to outcomes. Because food allergies vary in severity, aeNP treatment may lead to variation in the phase of immune regulation between individual mice. For example, in experimental autoimmune encephalomyelitis, depending on the milieu of B cells and disease initiation, repletion of B cells can have a more pathogenic phenotype versus a more naive phenotype (51, 52). Thus, B cell depletion during aeNP treatment of food allergies could induce opposing B cell phenotypes depending on the specific immune state of each mouse. Ultimately, B cells have complex roles in immune regulation, and the mechanism of aeNP immunomodulation cannot be explained by one cell type in isolation but rather the communication of many different cell types.

The mechanism of aeNP association to cells is preferential to a B cell–specific function or structure as indicated by biodistribution studies of i.v. aeNP delivery showing that a majority of aeNP^+^ cells are B cells, although aeNPs were not designed to target B cells specifically. Association of aeNPs with B cells appears to be allergen-agnostic, as the frequency of aeNP-associated B cells in the SILP was largely unchanged regardless of sensitization with peanut-alum–sensitized mice as well as healthy mice showing similar aeNP–B cell association. However, allergen-specific mechanisms may still be involved, as evidenced by the proportion of SILP OVA NP^+^ B cells relative to all NP^+^ cells increasing with the number of doses delivered in OVA-alum–sensitized mice. Analogously, a dose-dependent increase of OVA NP^+^ B cells, relative to all SILP B cells, in mice sensitized to OVA-alum and PE-alum compared with unsensitized mice was observed, suggesting a food allergic disease–specific mechanism. The aeNP–B cell association is preferential for B cells expressing CD23, a low-affinity IgE receptor, suggesting a possible mechanism of association is through antibodies binding allergen protein epitopes exposed on the surface of NPs, which then allow for binding to CD23. Previous studies have shown the necessity of allergen specificity for clinical efficacy as PLG (poly(lactide-*co*-glycolide)-COOH) NPs alone and NPs loaded with a nonspecific antigen do not reduce clinical symptoms of allergy (24, 55). Ultimately, this extensive association of aeNPs with B cells suggests a role for B cells in aeNP mechanism of action.

OVA NP treatment leads to the induction of Bregs, a B cell subtype shown to ameliorate allergic responses. Although some Breg subtypes in OIT (9, 10, 12, 17, 45) work through an allergen-specific isotype-switching mechanism, such as through generating allergen-specific neutralizing IgG4 antibodies, Bregs after aeNP treatment do not seem to have this function given that OVA-specific antibodies are unchanged after treatment. Other types of Bregs are thought to be able to expand Treg populations, suppress effector T cells, and downregulate costimulatory molecules on DCs through IL-10, TGF-β, and IL-35 cytokines and thrombospondin-1 secretion (12, 45). Analogously, B cells after OVA NP treatment have markers associated with Bregs found in mice and humans (36, 37). Specifically, OVA NP administration is associated with B cells upregulating CD29 in the spleen. CD29, also known as VLA-4, is an integrin on the surface of immune cells known to facilitate immune cell migration (38). CD29 has previously been used as a biomarker of efficacy for patients with grass pollen allergy undergoing SLIT, suggesting aeNP immunotherapy similarly skews pathogenic B cell phenotypes to more regulatory fates (46). Additionally, Breg markers associated with a type of IL-10–secreting Bregs, B10 cells, are expressed in both allergic and OVA NP–treated mice, yet the markers expressed are distinct, suggesting the functionality of the Bregs present in allergic mice is impaired. In alignment with this finding, we identified an IL-10–secreting B cell population in the SILP that showed more IL-10 expression and less MHC-II expression with OVA NP treatment. Receptor-ligand analysis also showed differences in IL-10 Breg cell crosstalk with other immune cell types after OVA NP treatment. Specifically, IL-10^+^ Bregs in OVA NP–treated mice are downregulating communication with other immune cells via PARs, CypA, and CLEC signaling, which is associated with allergy-driving immune activation (56, 57), and upregulated IL-10 and PECAM-1 signaling, associated with tolerogenic immune signaling (10, 40–43). These changes in signaling are similar to clinical findings in patients with cow’s milk allergy undergoing OIT: after OIT, allergen-specific B cells increase expression of the IL-10 receptor and downregulate B cell activation markers (9).

Further supporting the regulatory functions of B cells after aeNP treatment, we identified a disruption in regulatory signaling within the spleen and SILP with CD20^+^ B cell depletion during aeNP administration. Previously, we identified that aeNP efficacy depends on tolerogenic CD103^+^ DCs and reprogramming of Th2 T cells to regulatory fates (25). The increase in splenic CD103^+^ DCs after aeNP treatment was absent in CD20^+^ B cell–depleted mice, indicating CD103^+^ DC expansion has a CD20^+^ B cell–dependent component. Although B cells inducing tolerogenic CD103^+^ DCs is not well studied, B cells affecting DC function has been well documented (58–61). We anticipate CD23^+^ B cells are affecting CD103^+^ DCs through similar mechanisms such as B cell–derived cytokines and chemokines, contact-dependent signaling, or through signaling with an intermediary cell type that induces CD103^+^ DC signaling. Because CD103^+^ DCs induce Tregs with gut-homing markers (34, 35), the B cell–dependent increase in CD103^+^ DCs after OVA NP treatment could explain the B cell–dependent increase in CCR9 gut-homing marker expression on SILP Tregs. This result suggests B cells may have a role in inducing migration of Tregs to the SILP via increasing gut-homing marker expression via expansion of CD103^+^ DCs.

Consequently, the induction of Bregs by aeNP treatment contributes to distinct communication dynamics within the SILP that attenuates allergic responses. Allergy-driving signaling, such as IL-4 signaling, shows tolerogenic communication with OVA NP treatment. Although IL-4 signaling is present in both allergic PBS-treated and OVA NP–treated SILP, IL-4 signaling is changed with OVA NP treatment. Specifically, IgG1^+^ B cells in the SILP of OVA NP–treated mice do not participate in IL-4 signaling, unlike in allergic mice, suggesting that B cell allergy-driving pathways are being suppressed. Moreover, CD103^+^ DC IL-4 communication is only present with OVA NP treatment, suggesting tolerogenic cell communication is being induced.

Because OVA NPs show the greatest association with CD23-expressing B cells, and SILP B cells upregulate CD23 signaling after aeNP treatment and OFCs, we conjecture that OVA NP effects on B cells have a CD23-mediated component. CD23 has dual roles of inflammatory and noninflammatory signaling. As a low-affinity IgE receptor on B cells, CD23 can bind IgE-allergen complexes, which can lead to different immune responses including B cell antigen presentation leading to T cell activation, transepithelial transport of IgE-allergen complexes, IgE suppression and upregulation, and B cell recycling of CD23-IgE allergen complexes leading to APC antigen presentation (47, 62, 63). Specifically, in the OVA NP–treated SILP after OFC 7, CD23 (*Fcer2a*) on B cells is predicted to interact with CD11c (ITGAX_ITGB2) and CD11b (ITGAM_ITGB2) on APCs. Thus, OVA NP treatment appears to disrupt MHC-II–mediated B cell–T cell interactions and to induce CD103^+^ DC MHC-II signaling to T cells. Additionally, the IL-10^+^ Breg subset in OVA NP–treated mice upregulates this CD23 communication with CD103^+^ DCs and macrophages, indicating this crosstalk may be tolerogenic. A potential tolerogenic mechanism via CD23 signaling may be that OVA NP treatment induces a B cell recycling of CD23-IgE allergen complexes to APCs that leads to antigen presentation by CD103^+^ DCs that inhibits allergy-driving T cells and promotes Tregs. Consequently, CD23 signaling in B cells after OVA NP treatment seems to be contributing to regulatory functions in B cells and APCs.

Delineating whether aeNP modulation of the B cell compartment depends more on the direct association of aeNPs to B cells versus the indirect conditioning of the environment after aeNP treatment will require additional studies. The CD20^+^ B cell depletion study identified potential direct and indirect effects of aeNPs on B cells, but with limitations. Although anti-CD20 was effective at depleting CD20^+^ B cells across all tissues analyzed, other aeNP^+^B220^+^CD138^–^ B cells were present in the SILP. Because the SILP is the site of pathology and a location where we identified aeNP-induced changes to B cell communication, the presence of CD20^–^ B cells in the SILP may contribute to the incomplete break in efficacy of aeNPs after anti-CD20 administration. Although clinical outcomes were not significantly different with CD20^+^ B cell depletion, we identified a trend in more severe reactors after aeNP treatment and a strong negative correlation between serum MCPT-1 levels and anaphylaxis-induced body temperature drops that was not present in the control OVA NP–treated mice. Herein, the bulk RNA-Seq study was performed before OFCs, and the single-cell sequencing study was analyzed for allergic PBS-treated mice with severe allergic reactions. Future studies to parse differences in aeNP immunomodulation will need to be performed for varying severities of allergic disease.

Our studies demonstrate aeNP treatment induces Breg responses in a mouse model of food allergy. Because the role of B cells beyond secreting allergen-specific antibodies in food allergies is not fully understood, our work contributes to elucidating key B cell functions during an NP-based allergen-specific immunotherapy. Biodistribution studies showed that aeNPs readily associate with B cells across immune organs. Depleting CD20^+^ B cells during aeNP treatment identified that B cells contribute to splenic CD103^+^ DC expansion and SILP Treg gut-homing marker expression. RNA-Seq identified subtypes of B cells expressing Breg markers, including those from IL-10–secreting Bregs. Further single-cell analysis of B cell communication in the SILP identified CD23 signaling as a potential inducer of regulatory CD103^+^ DC functions and disruptor of pathogenic immune activation, including B cell–T cell crosstalk. Because aeNPs only associate with a small proportion of B cells overall, engineering aeNPs to further target B cell association may improve aeNP efficacy and further elucidate the mechanisms by which aeNPs induce Breg functions. Understanding these Breg mechanisms will help improve food allergy therapies and move toward inducing tolerance as opposed to desensitization.

## Methods

### Sex as a biological variable.

Our study exclusively examined female mice. It is unknown whether the findings are relevant for male mice.

### Animals.

Animal housing was temperature controlled with 12-hour light/12-hour dark cycles. Food and water were available ad libitum. For all experiments described below, female BALB/cJ mice (000651; The Jackson Laboratory) were utilized.

### NP fabrication and characterization.

A double-emulsion (w/o/w) solvent evaporation method was used to fabricate OVA (grade V with MW 44.3 kDa; Sigma-Aldrich) NPs, as previously described (24, 25, 64). In brief, 50:50 poly(lactide-*co*-glycolide)-COOH (PLG; i.v. = 0.18; Evonik) was dissolved at 20% w/v in 2 mL of dichloromethane. Next, 150 μL of 200 mg/mL OVA in PBS was added to the PLG solution and emulsified by sonication at 100% amplitude for 30 seconds, followed by the addition of 10 mL of 2% w/v aqueous (MW 400 kDa) poly(ethylene-alt-maleic anhydride) (PEMA; Polysciences, Inc.) as a surfactant stabilizer sonicated at 100% amplitude for 30 seconds. The resulting emulsion was stirred overnight in a 0.5% w/v PEMA solution. To remove surfactant, NPs were washed 4 times by centrifugation (5,000*g*, 15 min, 4°C) in 0.1 M sodium bicarbonate–sodium carbonate buffer, pH 9.6 (Polysciences, Inc.), and lyophilized in 3% w/v aqueous D-mannitol and 4% w/v aqueous sucrose. NPs labeled with Cy5.5 (Lumiprobe) were made using 1% w/w Cy5.5-poly(lactide-*co*-glycolide) synthesized using EDCI chemistry as previously described (32). A Zetasizer Nano ZSP was used to measure size by dynamic light scattering and charge by zeta potential analysis of OVA NPs in Milli-Q water to confirm 400–700 nm diameter, polydispersity index of less than 0.3, and surface zeta potential of less than −35 mV. A Micro BCA assay (Thermo Fisher Scientific) following the manufacturer’s instructions using an OVA standard curve was used to confirm allergen loading of 40 μg OVA/mg of NP after dissolving NPs in sodium hydroxide. Flow cytometry for OVA was done to show the percentage of OVA NPs that contain OVA. In brief, OVA polyclonal antibody (Invitrogen, PA1-196; 1:100 dilution) was incubated with 0.1 mg of NPs for 15 minutes at room temperature, followed by incubation with donkey anti-rabbit IgG H&L Alexa Fluor 405 (Abcam, Ab175651; 1:100 dilution) for 15 minutes at room temperature.

### Murine egg allergy model.

Mice were sensitized to OVA as previously described (25). In brief, on days 0 and 14, 4- to 6-week-old mice were sensitized i.p. using 20 μg OVA adsorbed to 1 mg of aluminum hydroxide (alum; Alhydrogel adjuvant 2%, InvivoGen). On day 27, saphenous bleeds were performed to assess serum OVA-specific IgE and IgG1 titers (as described below); mice with IgE titers of 200 or less and IgG1 titers of 50,000 or less were excluded from experimentation for incomplete sensitization. On days 28 and 42, OVA NPs were given i.v. at 2.5 mg per 100 μL dose. OVA NPs were washed in PBS before administration. Controls were given i.v. 100 μL of PBS. OFCs were done 7 times over the course of 2 weeks. OFCs were done on days 45, 47, 49, 52, 54, 56, and 59. OFCs involved intragastric (i.g.) delivery of 10 mg OVA in PBS after a 5-hour fast. Saphenous bleeds were done 30–60 minutes after OFC 7 to measure serum MCPT-1 levels. Cardiac punctures for blood collection were done to measure serum OVA-specific IgE, IgG2a, and IgG2b antibody titers, 24 hours after OFC 7. As previously described, ELISA was used to measure OVA-specific antibody titers (65, 66) and MCPT-1 (66, 67). After i.g. OVA administration, clinical outcomes after OFC 6 and OFC 7 were assessed by measuring rectal temperature and evaluating behavioral symptoms every 15 minutes for 1 hour (68–70). Clinical scores were as follows: 0 = no symptoms; 1 = scratching; 2 = diarrhea, reduced activity, hunched posture, scruffy fur; 3 = difficulty breathing; 4 = laying flat with little to no movement; 5 = death. Mice with body temperatures of less than 33°C were placed on a heating pad set to 37°C under an infrared heat lamp and s.c. administered 1 mL of lactated ringers.

### Tissue harvest and processing.

Mesenteric lymph nodes, Peyer’s patches, spleens, and the proximal 15 cm of the small intestine (duodenum and jejunum) were harvested and processed into single-cell suspensions as previously described (25). In brief, mesenteric lymph nodes were mechanically dissociated through a 70 μm filter and were then ready for further analysis. Spleens were mechanically dissociated through a 70 μm filter, subjected to ACK lysis for 2–5 minutes, filtered again through a 70 μm filter, and were then ready for further analysis. SILP were processed through de-epithelialization by 4 washes in 5 mM EDTA in HBSS and minced. Minced SILP and Peyer’s patches were digested in a buffer of 2 mg/mL, collagenase IV (Sigma-Aldrich), and 0.20 mg/mL DNase I (Sigma-Aldrich) in RPMI 1640 containing 10% heat-inactivated FBS (VWR) at 37°C for 20 minutes or 30 minutes, respectively, on a shaker. After digestion, SILP and Peyer’s patches were mechanically dissociated through a 19-gauge needle and filtered through a gauze filter or a 70 μm filter, respectively, to generate single-cell suspensions for further use. SILP lymphocytes were enriched using a Percoll gradient of 44% to 67% in RPMI, and the cells from the interface of the 2 solutions were collected for downstream analysis.

### B cell depletion during OVA NP treatment.

Mice were sensitized on days 0 and 14 with 1 mg alum plus 20 μg OVA (i.p.). One week before each OVA NP dose, 250 μg i.v. of murine anti-CD20 (Bio X Cell; BE0356) was used to deplete mature B cells, and controls were given 250 μg i.v. of a nonreactive murine antibody (Bio X Cell; BE0093) (71). OVA NPs were given i.v. on days 49 and 63. OFCs with 10 mg OVA i.g. were done on days 66, 68, 70, 73, 75, 77, and 80. Serum- specific OVA titers were done 24 hours before the first OVA NP dose and 24 hours after OFC 7 as described above in *Murine egg allergy model*. Serum MCPT-1 levels were measured after OFC 7 as described above in *Murine egg allergy model*. Splenic DCs and SILP T cells were phenotyped by flow cytometry on day 81. Clinical outcomes were stratified based on allergic reaction severity. No reactivity was denoted as having a temperature reduction of less than 1°C or having a clinical score of 0 and with an absence of diarrhea. A mild reactivity was denoted as having a temperature reduction between 1°C and 2.5°C or a clinical score of 1 to 2 or having diarrhea. Severe reactivity was characterized as a temperature reduction of greater than 2.5°C or a clinical score greater than 2.

### Biodistribution of OVA NPs.

Mice were sensitized on days 0 and 7 i.p. with 1 mg alum plus 20 μg OVA. Cy5.5-labeled OVA NPs were given i.v. on days 14 and 21. Peyer’s patches, mesenteric lymph nodes, SILP, and spleens were harvested 24 hours after each OVA NP dose on days 15 and 22. Cy5.5-NP^+^ immune cells were identified by flow cytometry as described in *Flow cytometry*. For biodistribution studies with anti-CD20 (Bio X Cell; BE0356), mice were sensitized i.p. with OVA/alum (days 0 and 7) and given anti-CD20 on day 14. On day 21, all mice received treatment with 2.5 mg of i.v. Cy5.5-conjugated OVA NPs. Peyer’s patches, mesenteric lymph nodes, SILP, and spleens were harvested on day 22, and Cy5.5-NP^+^ immune cells were identified by flow cytometry as described in the following section.

### Flow cytometry.

All conventional flow cytometry studies were done using a ZE5 Cell Analyzer (Bio-Rad), and FlowJo was used for analysis. The spleen DC panel included CD11c (BV605; BioLegend, 117334), CD103 (APC; BioLegend, 121413), CD11b (PE; BioLegend, 101208), MHC-II (FITC; 107605), CD45 (AF700; BioLegend, 157615), F4/80 (PE/Cy7; BioLegend, 123113), and DAPI (BioLegend). The SILP T cell panel included Live/Dead Fixable Violet (Thermo Fisher Scientific, L34955), CD25 (BV605; BioLegend, 102036), CD4 (FITC; BioLegend, 100510), CCR9 (PerCP-eFluor 710; Thermo Fisher Scientific, 46-1991-82), CD8 (PE/Cy7; BioLegend, 100722), LPAM-1 (APC; Miltenyi Biotec, 130-123-565), and FoxP3 (PE; BioLegend, 126404). A True-Nuclear Transcription Factor buffer set (BioLegend) was used for intracellular FoxP3 staining following the manufacturer’s instructions with rat serum (Invitrogen) used for blocking nonspecific binding before staining. Cell surface staining was done after anti-CD16/32 for Fc-blockade.

Three panels were used for the OVA NP biodistribution study in mice sensitized with OVA-alum, PE-alum, and PBS (control). Panel 1 comprises CD11c (BV605; BioLegend, 117334), CD11b (PerCP-Cy5.5; BioLegend, 101227), F4/80 (PE/Cy7; BioLegend, 123113), CD103 (FITC; BioLegend, 121419), and Live/Dead Fixable Violet (Thermo Fisher Scientific, L34964). Panel 2 comprises CD4 (BV421; BioLegend, 100543), CD8 (FITC; BioLegend, 100705), B220 (PerCP; BioLegend, 103234), CD138 (PE/Cy7; BioLegend, 142514), and Live/Dead Fixable Violet (Thermo Fisher Scientific, L34964). Panel 3 comprises FcεRI (FITC; Invitrogen, 11-5898-82), CD117 (BV605; BioLegend, 135121), CD200R (PE-Vio 770; Miltenyi Biotec, 130-112-532), Siglec-F (PE; Invitrogen, 12-1702-80), CD11b (PerCP/Cy5.5; BioLegend, 101227), and Live/Dead Fixable Violet (Thermo Fisher Scientific, L34964).

Biodistribution of OVA NPs with anti-CD20 was done using spectral flow cytometry using a Cytek Aurora cytometer. The panel included CD45R (B220) (Spark UV 387; BioLegend, 103296), CD138 (APC-Cy7; BioLegend, 142530), CD23 (Brilliant Violet 785; BioLegend, 101645), CD11b (PE-Fire 810; BioLegend, 101285), F4/80 (PE/Dazzle 594; BioLegend, 123146), CD11c (BV650; BioLegend, 117339), CD103 (BV421; BioLegend, 121422), FceR1 (FITC; BioLegend, 134305), CD117 (BV605; BioLegend, 135121), Siglec-F (PE; Invitrogen, 12-1702-80), CD200R (PE-vio 770; Miltenyi Biotec, 130-112-532), CD20 (APC-Fire 810; BioLegend, 150438), and Zombie UV Fixable Viability kit (BioLegend, 423108).

IL-10 B cell flow cytometry was done using spectral flow cytometry using a Cytek Aurora Evo cytometer. The panel included CD45R (B220) (Spark UV 387; BioLegend, 103296), CD138 (APC-Cy7; BioLegend, 142530), MHC II (FITC; BioLegend, 107606), IL-10 (PE/Dazzle 594; BioLegend, 505034), and Zombie UV Fixable Viability kit (BioLegend, 423108). Prior to surface and intracellular cytokine staining, single-cell suspensions were stimulated for 4 hours in B cell medium with 50 μg/mL PMA, 1 μg/μL ionomycin (Cell Activation Cocktail; BioLegend, 423302), and protein transport was inhibited using 2 μM monensin (BioLegend, 420701). B cell medium comprised 2 mM L-alanyl-L-glutamine (1× Gibco GlutaMAX), 10% FBS, 10 mM HEPES pH 7.5, 100 U/mL penicillin and 100 μg/mL streptomycin, and 100 μM 2-mercaptoethanol in advanced RPMI 1640 (Invitrogen, 12633012) culture medium. Intracellular cytokine staining was done using Cyto-Fast Fix/Perm buffer set (BioLegend, 426803) following the manufacturer’s instructions.

### Bulk RNA-Seq of splenic and Peyer’s patch B cells.

Mice were sensitized on days 0 and 14 i.p. with 1 mg alum plus 20 μg OVA, and then mice were treated with i.v. OVA NPs on days 28 and 42. On day 44, B cells from the spleen and Peyer’s patches were isolated using magnetic activated cell sorting by negative selection using the Pan B Cell Isolation kit II (Miltenyi Biotec, 130-104-443) following the manufacturer’s instructions. Cells were resuspended in TRIzol Reagent (Invitrogen) before isolating RNA using Direct-zol RNA Miniprep Plus (Zymo) following the manufacturer’s instructions. B cell RNA was submitted for 151 bp paired-end sequencing according to the manufacturer’s protocol (Illumina NovaSeq). De-multiplexed FastQ files were made using BCL Convert Conversion Software v4.0 (Illumina). Cutadapt v2.3 was used to trim reads (72). Data quality was assessed using FastQC (v0.11.8) and FastQ Screen (v0.15.3) (73). Reads were mapped using STAR (v2.7.8a) to reference genome GRCm38 (ENSEMBL 102), and genes were assigned count estimates with RSEM (v1.3.3) (74). ENCODE standards for RNA-Seq were followed. QC metrics were aggregated by multiQC (v1.7) (75).

DESeq2 was used for differential expression analysis (76). DEGs were found per tissue between OVA NP–treated and PBS-treated mice with a log fold-change cutoff of 0.25 and adjusted *P* value cutoff of 0.05. To rank the DEGs by log fold change, the shrinkage of effect size (log fold change) was done using an adaptive normal distribution as a prior distribution (76).

Breg markers were identified in the literature (36, 37), which were defined as DEGs compared with other B cell subsets. Specifically, Yang et al. identified Breg markers to include upregulated and downregulated markers from well-defined Breg subsets such as IL-10–secreting B10 cells compared with other B cell subsets (11, 36) as well as Breg genes that are common across the liver, spleen, bone marrow, peritoneal cavity, and mesenteric lymph nodes (36). Additionally, Dubois et al. preformed a meta-analysis of mouse Breg datasets to identify Breg DEGs compared with non-Breg B cells (37). These markers were compiled and used for Breg identification in the DEGs between the OVA NP and PBS conditions. For visualization, the data were transformed by the regularized logarithm transformation using the *rlog* function in DESeq2. The CRAN package pheatmap (77) was used to visualize Breg markers differentially expressed between the OVA NP and PBS conditions.

### Functional enrichment analysis.

Gene ontology enrichment analysis was done using clusterProfiler (78). Overrepresentation analysis of gene ontology terms from the biological processes class was done using an input of a log fold-change ranked list of all genes with an adjusted *P* value of less than 0.05 as determined by DESeq2 per condition (PBS and OVA NP). Pairwise similarity of enriched terms was calculated using the Jaccard’s similarity index using the *pairwise_termism* function. Hierarchical clustering of enriched terms was done using the weighted pair group method with arithmetic means and plotted using the *treeplot* function.

### Single-cell RNA-Seq of SILP.

Mice were sensitized with 1 mg alum plus 20 μg OVA (i.p.) on days 0 and 14 and treated with OVA NPs or PBS on days 28 and 42. SILP were harvested from mice treated with OVA NPs and PBS after OFC 7 on day 60. SILP were processed into single cells. Each sample was pooled from 3–4 biological replicates. Allergic PBS-treated mice had anaphylaxis-induced temperature drops of 5°C or more, diarrhea, and serum MCPT-1 levels greater than 100 μg/mL; OVA NP–treated mice had temperature drops of 0.5°C or less, no diarrhea incidence, and serum MCPT-1 levels of less than 15 μg/mL. To sequence with enough representation of other immune cell types, two-thirds of B cells were depleted using a positive selection CD19 MicroBeads kit (Miltenyi Biotec, 130-121-301) following the manufacturer’s instructions. Dead cells were removed using the Dead Cell Removal kit (Miltenyi Biotec, 130-090-101). Cells were then fixed and permeabilized at 4°C for 24 hours using Chromium Next GEM Single Cell Fixed RNA Sample Preparation kit (10x Genomics, 1000414) following the manufacturer’s instructions. Samples were stored in 50% glycerol and Enhancer (10x Genomics, PN-2000482) at –80°C. Samples were library prepped following the manufacturer’s instructions for Chromium Fix RNA Profiling (10x Genomics) using the Chromium Mouse Transcriptome Probe Set (v1.0.1). This pool was subjected to 28 × 90 bp of sequencing according to the manufacturer’s protocol (Illumina NovaSeq XPlus). BCL Convert Conversion Software (v4.0, Illumina) was used to generate de-multiplexed Fastq files. Cell Ranger (v8.8.0) was used to align reads to mouse reference GRCm38 (mm10-2020-A).

SoupX was used to remove contaminating ambient RNA (79). Doublets were removed using DoubletFinder (80). Quality control was done with ddqc (81). Seurat (v5.1.0) (82) was used for cell clustering. All time points and treatment conditions were normalized and variance stabilized by SCTransform normalization and integrated via an anchor-based CCA integration. The data were visualized using uniform manifold approximation and projection for dimension reduction (UMAP). Cell types were annotated using markers identified using the FindConservedMarkers function. B cell subsets were identified based on expression of common B cell markers. Gene set enrichment analysis of the B cell subset was done using the SeuratExtend package using the GeneSetAnalysisReactome function on the immune system category of the Reactome gene set (83). The data were plotted using the WaterfallPlot function. CellChat (v2) was used for cell-cell communication analysis (39). The relative number of interactions was ranked between OVA NP and PBS SILP using the RankNet function. Differential expression analysis was used to compare OVA NP and PBS SILP communication such that any one of the subunits in the receptor/ligand complex could be differentially expressed with a log fold cutoff of 0.05.

### Statistics.

All statistical analysis was done in R (v4.3.0.). Type III 2-way ANOVA was used for analysis of flow cytometry using the car package (84) ANOVA function, and the emmeans package (85) was used to perform a post hoc Šidák’s test. Correlation tests were done using the cor.test function (stats package v4.3.0) based on the Pearson’s product moment correlation coefficient to test the null hypothesis that there is no correlation between paired samples. Linear regression line and 95% CI were calculated using geom_smooth in ggplot2 (v 3.5.2). Mann-Whitney *U* and 2-sided Student’s 2-tailed *t* tests were done using the ggpubr R package using the compare_means function with an α value of 0.05 considered significant (86). DEGs were identified using default parameters in DESeq2 such that Wald’s tests were done with *P* values adjusted using the Benjamini and Hochberg correction, with an α value of 0.05, and outliers were removed using Cook’s distance with a 0.99 quantile cutoff (87). Overrepresentation analysis of gene ontology terms was done in clusterProfiler by calculating the hypergeometric distribution, the same as a 1-sided Fisher’s exact test, to determine the *P* values with an α value of 0.05, and *P* values were adjusted using the Benjamini-Hochberg procedure. Statistical analysis done in CellChat used the default function parameters unless otherwise stated. The average mean for each cell group was calculated using the trimean method such that average gene expression was set to 0 if the percentage of cells expressing that feature was less than 25%. Significance between the number of interactions per signaling pathway was calculated via a paired Wilcoxon’s test with *P* less than 0.05. Differential expression analysis for signaling pathways between conditions was done by comparing communication probability using an ultra-fast Wilcoxon’s test using Presto (88).

### Study approval.

All procedures were performed in accordance with the regulations approved by the IACUC of the University of Michigan (protocol approval PRO00011621).

### Data availability.

Bulk RNA-Seq and single-cell RNA-Seq data are available from NCBI’s Gene Expression Omnibus (GEO GSE301060 and GSE300948, respectively). The Supporting Data Values file provides the data values for all graphs and values behind any reported means in the manuscript or supplemental materials. Raw data and analysis files associated with this manuscript are available in the Deep Blue Data Repository (89).

## Author contributions

LMR, SDM, LDS, and JJO contributed to conceptualization. LMR and MNS contributed to methodology. LMR, MNS, LAW, KWJ, KVG, EJB, JAM, SAT, JJO, CD, and JR contributed to investigation. LMR, LDS, and JJO contributed to visualization. LDS and JJO contributed to supervision. LMR, SDM, LDS, and JJO contributed to writing of original draft. LMR, EJB, LDS, and JJO contributed to writing related to revisions and editing.

## Conflict of interest

LDS consults for and has financial interests in Cour Pharmaceutical Development Company Inc., which has licensed the nanoparticle technology described in this manuscript.

## Funding support

This work is the result of NIH funding, in whole or in part, and is subject to the NIH Public Access Policy. Through acceptance of this federal funding, the NIH has been given a right to make the work publicly available in PubMed Central.

- NIH grants R01AI155678 (to LDS, JJO, and SDM) and T32GM007863 (to MNS).
- National Cancer Institute at the NIH award P30CA046592 (to The University of Michigan Advanced Genomics Core and the UM Single Cell Spatial Analysis Program for the use of the following: University of Michigan Cancer Center Shared Resource, the Single Cell and Spatial Analysis Shared Resource).

## Supplementary Material

Supplemental data

Supporting data values

## Version 1 06/09/2026

In-Press Preview

## Version 2 08/10/2026

Electronic publication
