# Supplementary material for: Regulatory B cells contribute to allergen-encapsulating nanoparticle immunotherapy efficacy for food allergy
Source: JCI Insight. 2026 Jun 9;11(15):e199988. doi: 10.1172/jci.insight.199988 (PMC13463620; doi:10.1172/jci.insight.199988)
Supplement: Supplementary file 1 [file jciinsight-11-199988-s201.pdf]

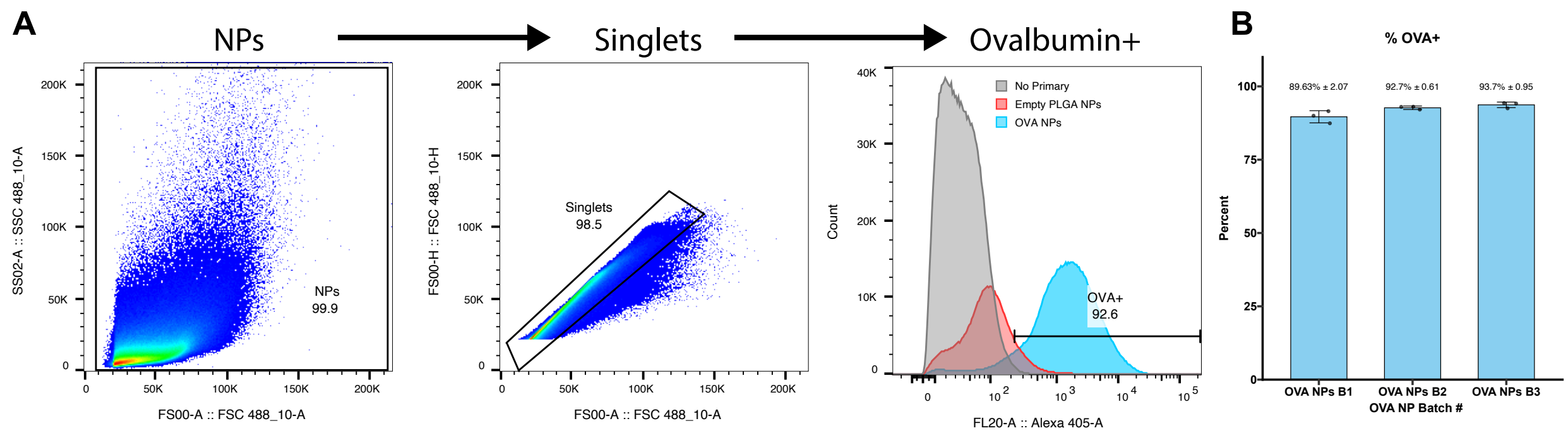

Supplemental Figure 1. *Flow cytometry for ovalbumin in ovalbumin-encapsulating nanoparticles (OVA NPs).* Flow cytometry gating scheme to identify OVA positive nanoparticles (**A**). Nanoparticles were gated using forward scatter area and side scatter area. Singlets were identified through a forward scatter area by forward scatter height gate. Then, OVA positive nanoparticles were identified of singlets. The histogram of OVA positive nanoparticles shows a no primary control of OVA NPs stained with AF405 secondary (grey) as well as PLGA nanoparticles that contain no protein (red). Percent of OVA positive nanoparticles of all singlets for three different batches of OVA NPs with 3 technical replicates per batch. Error bars indicate mean ± SD.

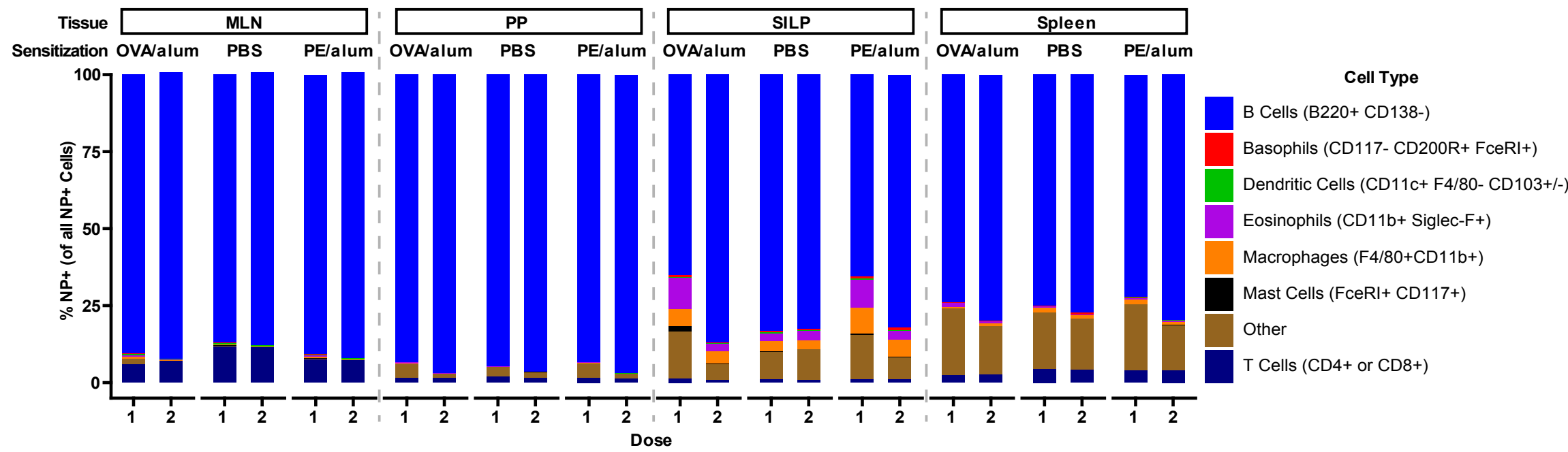

Supplemental Figure 2. *Biodistribution of OVA NPs in OVA allergic, peanut allergic, and naïve mice.* On days 0 and 7, BALB/cJ mice were sensitized i.p. with either OVA and alum, peanut extract (PE) and alum, or controls were given PBS. All mice received treatment with 2.5 mg of i.v. Cy5.5-conjugated OVA NPs on days 14 and 21. Mesenteric lymph nodes (MLNs), peyer's patches (PP), small intestine lamina propria (SILP), and splenocytes were isolated and analyzed via flow cytometry on days 15 and 22 to identify OVA NP-associated cells.

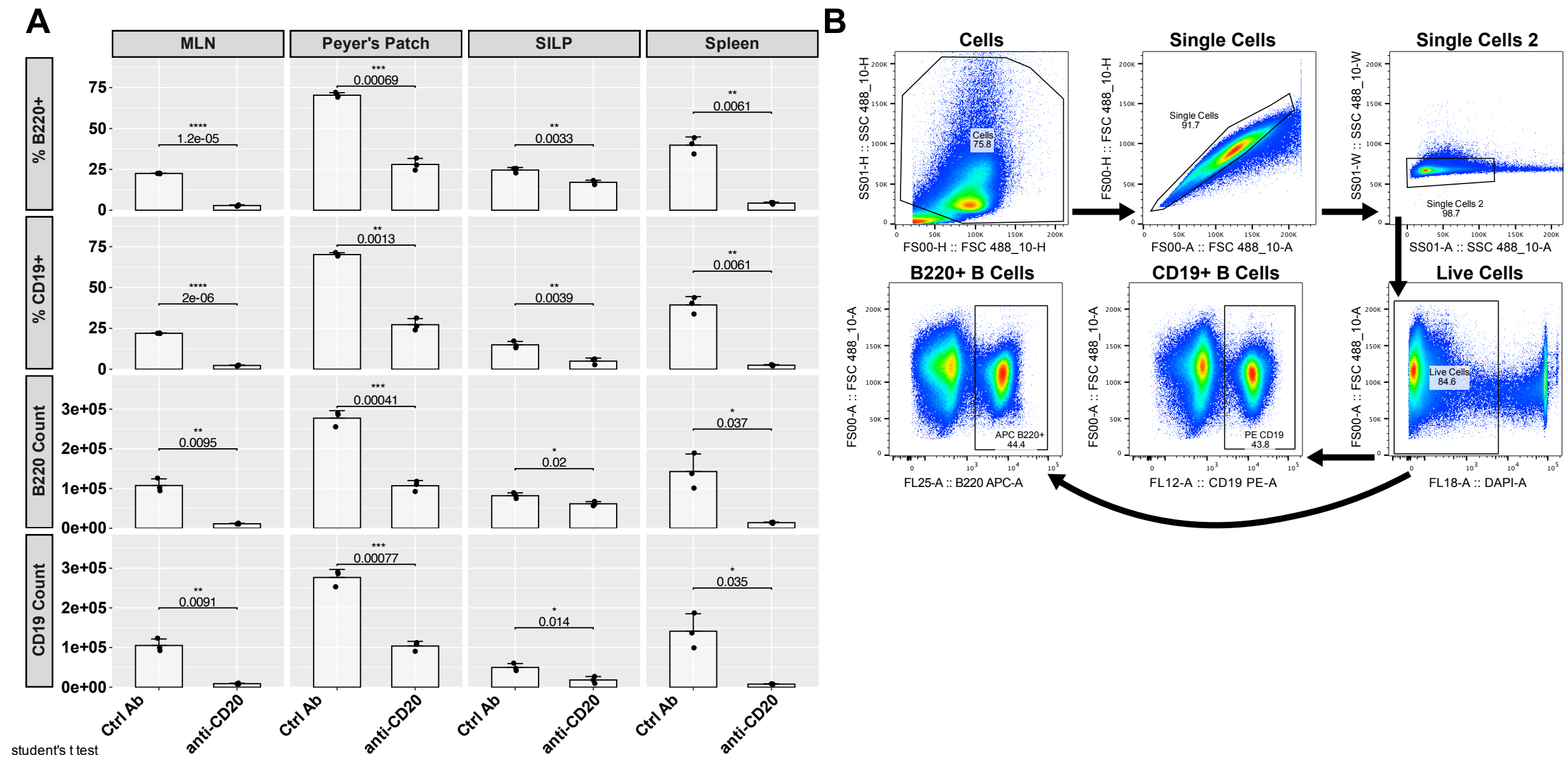

Supplemental Figure 3. *B cell depletion using anti-CD20 in allergic mice.* Mice sensitized i.p. with OVA-alum on days 0 and 14**,** were given anti-CD20 or control antibody on day 28. On day 35, flow cytometry for B cells using pan-murine B cell marker B220 or B cell-lineage marker CD19 was done in the spleen, Peyer's patches, mesenteric lymph nodes (MLN) and small intestine lamina propria (SILP) (Error bars indicate mean ± SD; n = 3 per group; student's t test with unequal variance) **(A)**. Gating scheme for B cell flow cytometry. Cells were gated using forward scatter height and side scatter height. Singlets were identified through two gates: a forward scatter area by forward scatter height gate followed by a side scatter area by side scatter width gate. Live cells were identified as DAPI negative by forward scatter area. B cells were identified as either CD19+ or B220+ using gates CD19 PE area or B220 APC area by forward scatter area, respectively **(B)**.

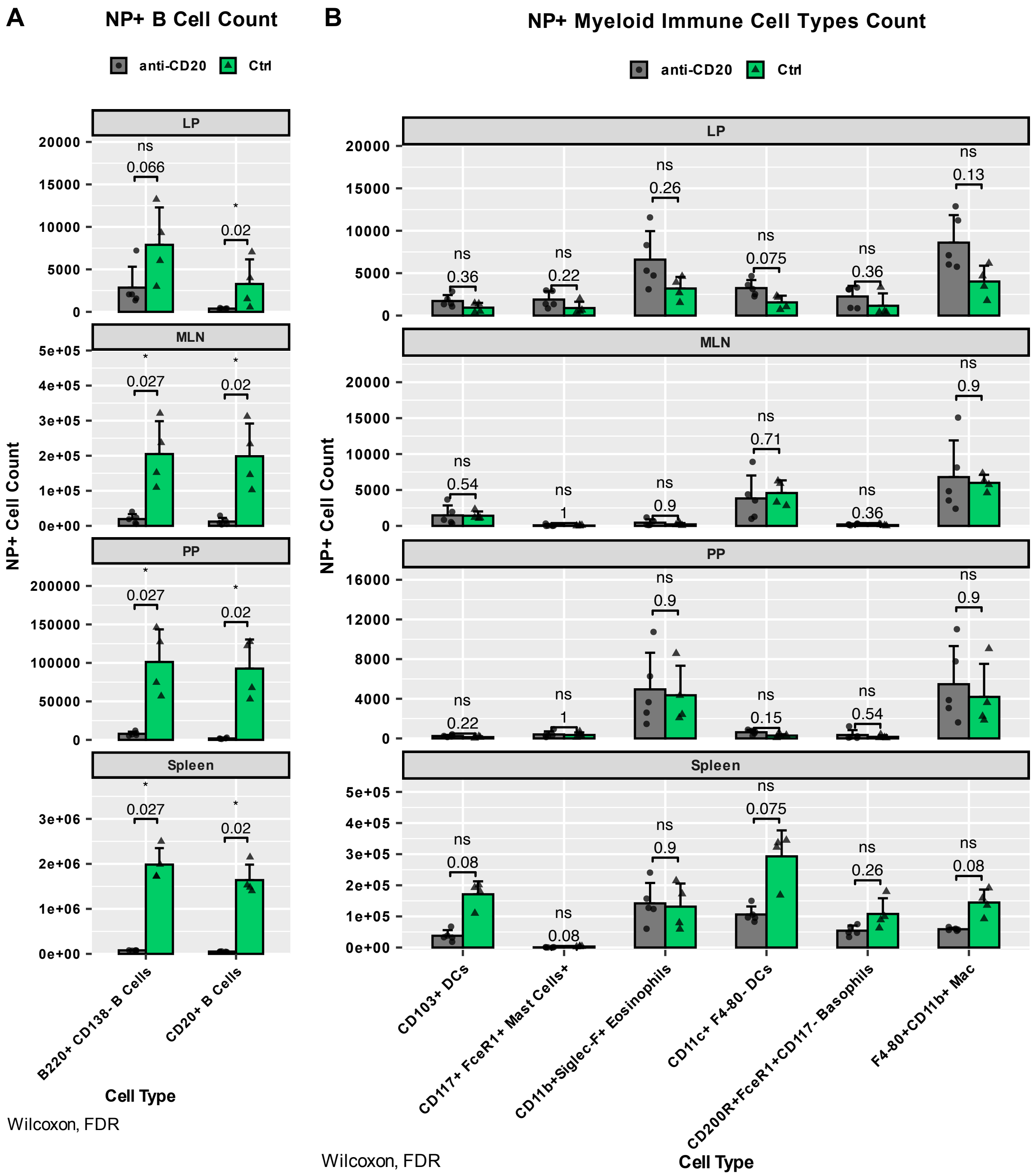

Supplemental Figure 4. *OVA NP biodistribution in OVA allergic mice with anti-CD20 B cell depletion.* Mice sensitized i.p. with OVA/alum (days 0 and 7) were given anti-CD20 on day 14. On day 21, all mice received treatment with 2.5 mg of i.v. Cy5.5-conjugated OVA NPs. Mesenteric lymph nodes (MLNs), peyer's patches (PP), small intestine lamina propria (SILP), and splenocytes were isolated and analyzed via flow cytometry on day 22 to identify OVA NP-associated cells. Counts for NP+ B cells with and without B cell depletion are shown **(A)**. There is not an appreciable amount of NP+ CD20+ B cells in the B cell-depleted group. NP+ cell counts for myeloid immune cell types **(B)** OVA NP biodistribution does not significantly change with B cell depletion (Error bars indicate mean ± SD; n = 4 in control and n = 5 in anti-CD20 group; multiple Wilcoxon tests with FDR correction).

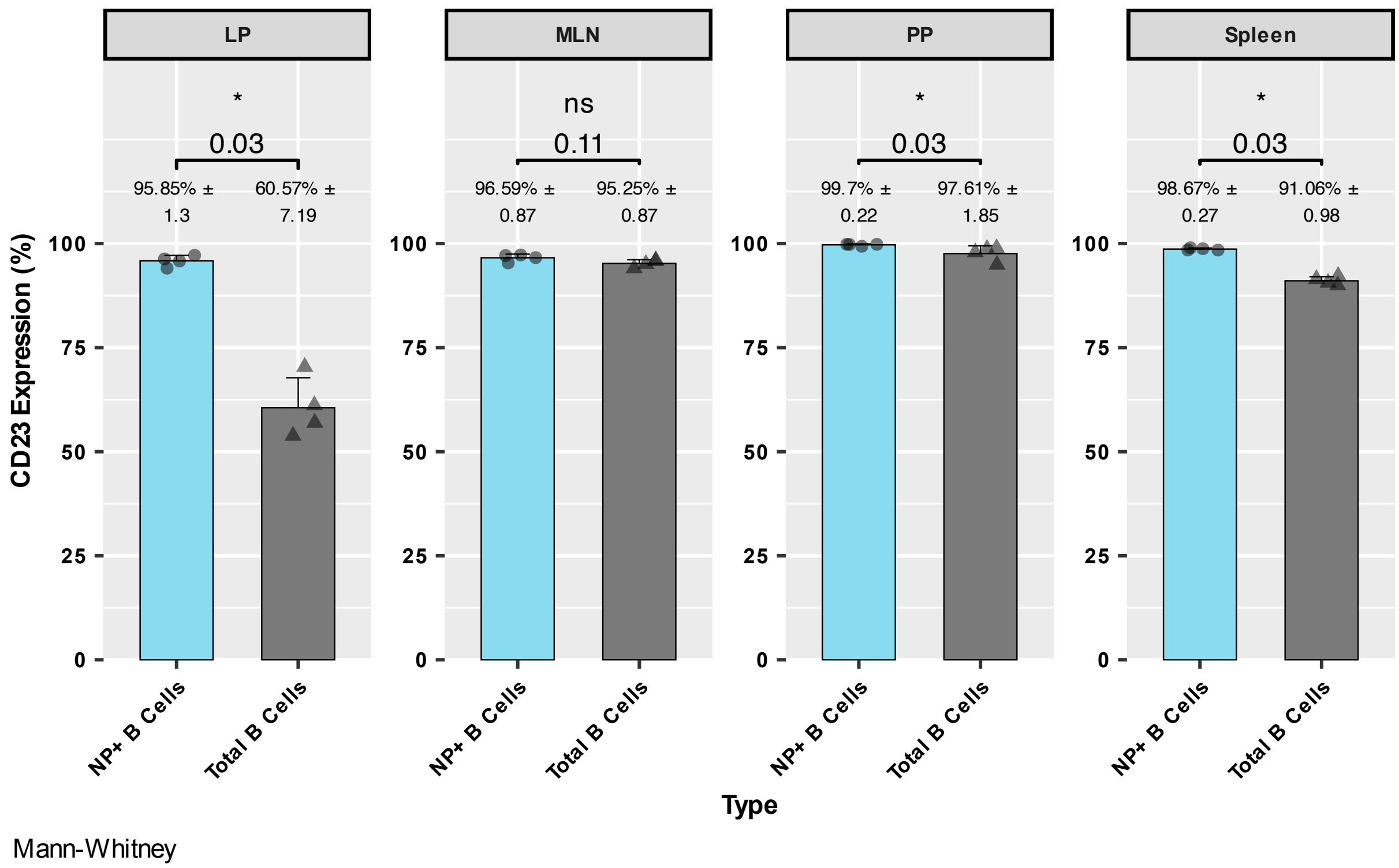

Supplemental Figure 5. *OVA NPs associate preferentially with CD23+ B cells.* Mice sensitized i.p. with OVA/alum (days 0 and 7) were 2.5 mg of i.v. Cy5.5-conjugated OVA NPs on day 21. Mesenteric lymph nodes (MLNs), peyer's patches (PP), small intestine lamina propria (SILP), and splenocytes were isolated and analyzed via flow cytometry on day 22 to identify OVA NP-associated cells. Percent of NP+ B cells expressing CD23 and percent of total B cells expressing CD23 is shown for all tissues. Error bars indicate mean ± SD; n = 4; Mann-Whitney

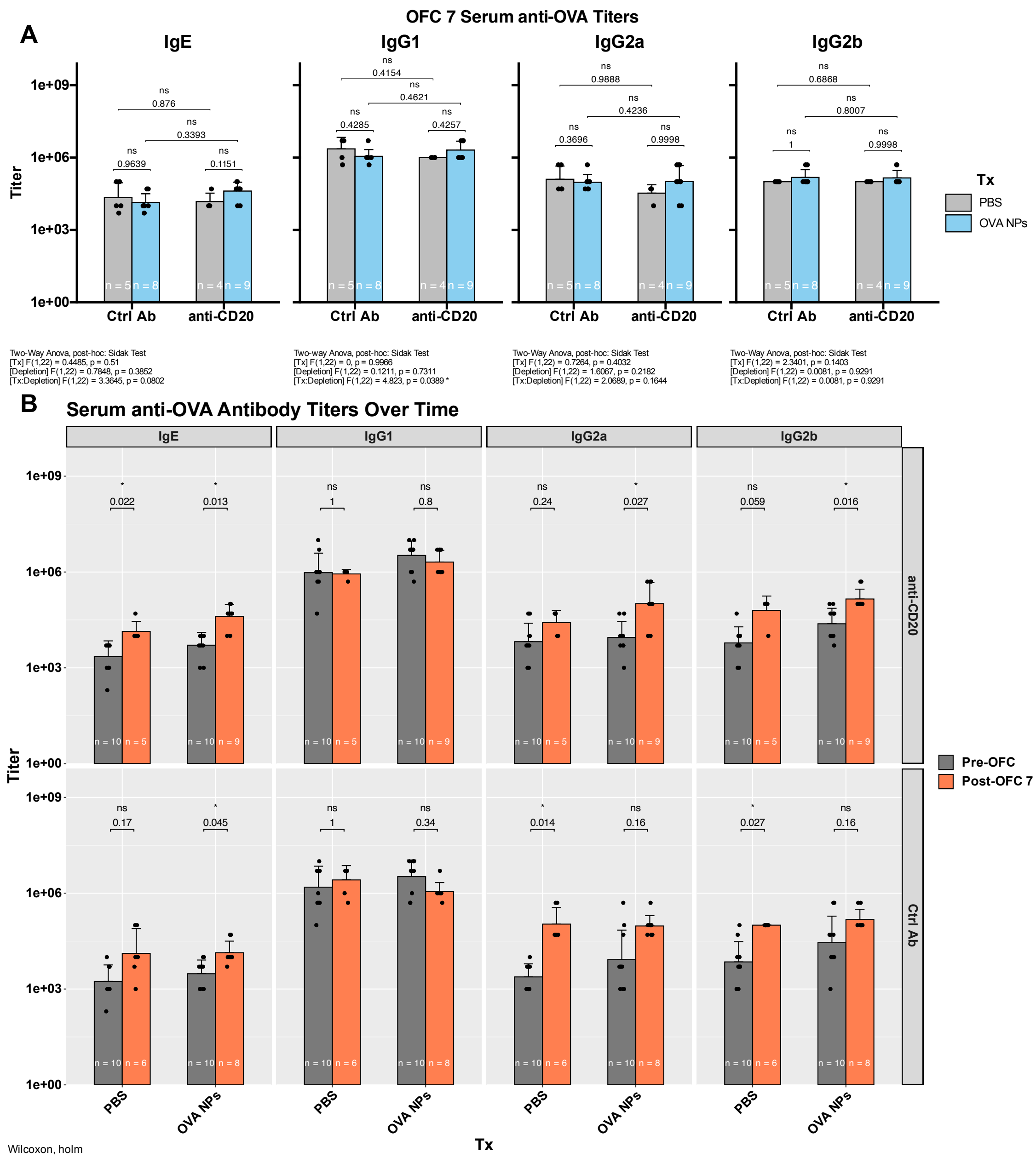

Supplemental Figure 6. *OFC 7 Serum anti-OVA titers in allergic and OVA NP-treated mice with anti-CD20 B cell depletion.* For the B cell depletion cohort (Figure 2A), serum was collected for anti-OVA antibody titers on day 41, before treatment or B cell-depleting antibody administration, and day 81, 24 hours after OFC 7. On day 81, after OFC 7, IgE, IgG1, IgG2a, and IgG2b titers did not change with anti-CD20 depletion or treatment **(A)**. Error bars indicate mean ± SD; statistically significant differences were identified using a two-way ANOVA with a post-hoc Sidak Test with p-values printed above significance brackets and sample size printed at the bottom of each bar. Changes in serum OVA-specific IgE, IgG1, IgG2a, and IgG2b titers from day 41 to day 81 was determined to be statistically significant using multiple Wilcoxon tests with holm adjustment (Error bars indicate mean ± SD) **(B)**.

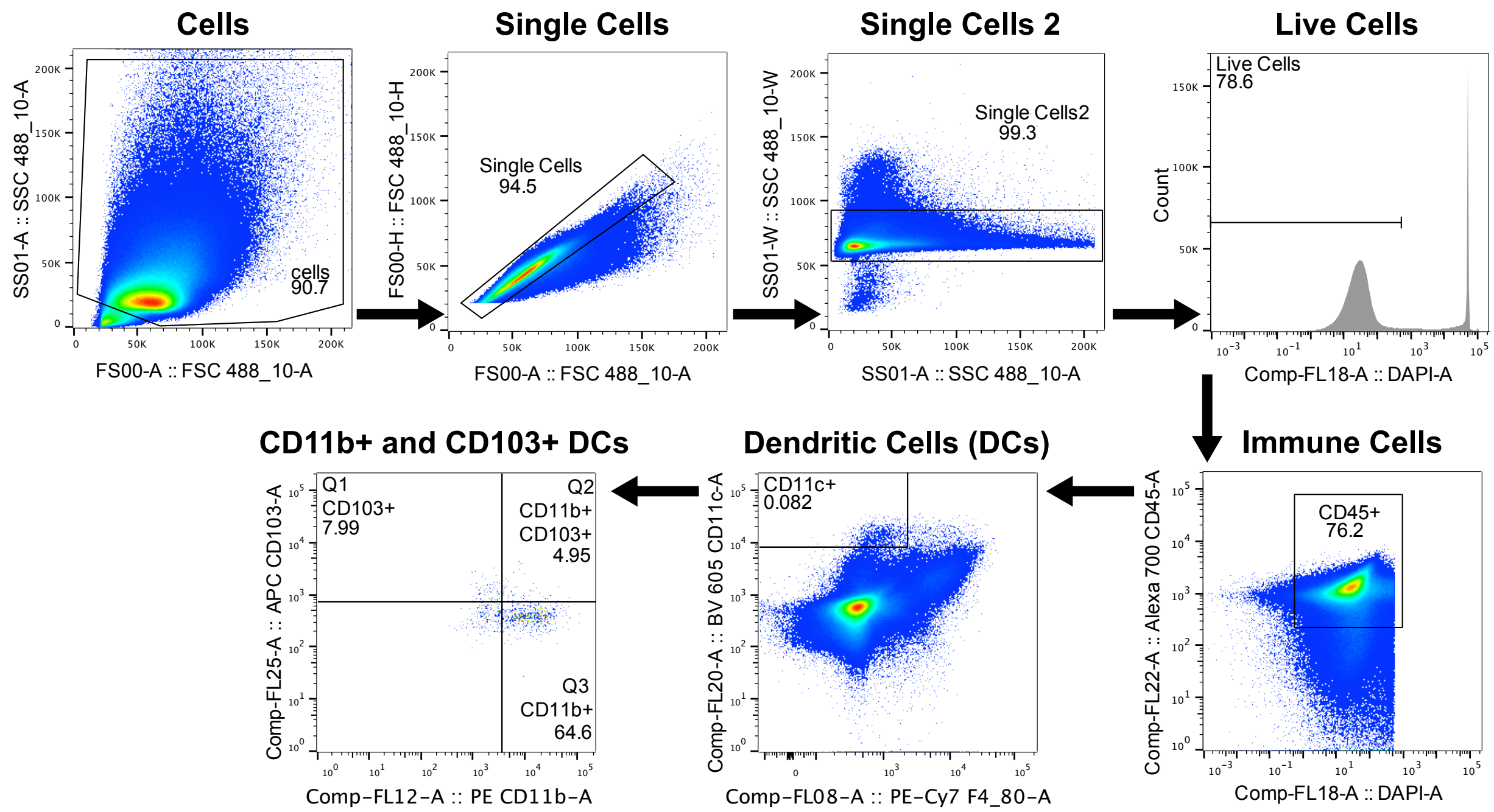

Supplemental Figure 7. *Gating scheme for CD103+ DC flow cytometry.* Cells were gated using forward scatter area and side scatter area. Singlets were identified through two gates: a forward scatter area by forward scatter height gate followed by a side scatter area by side scatter width gate. Then, live cells were identified as DAPI negative using a histogram. Of live cells, immune cells were identified as CD45+ using a gate in DAPI area by AF700 area. Of immune cells, dendritic cells were identified as CD11c+ F4/80-. CD103+ DCs were defined as CD103+ (Q1) or CD103+ CD11b+ DCs (Q2). CD11b+ DCs were defined as CD11b+ (Q3) or CD11b+ CD103+ DCs (Q2).

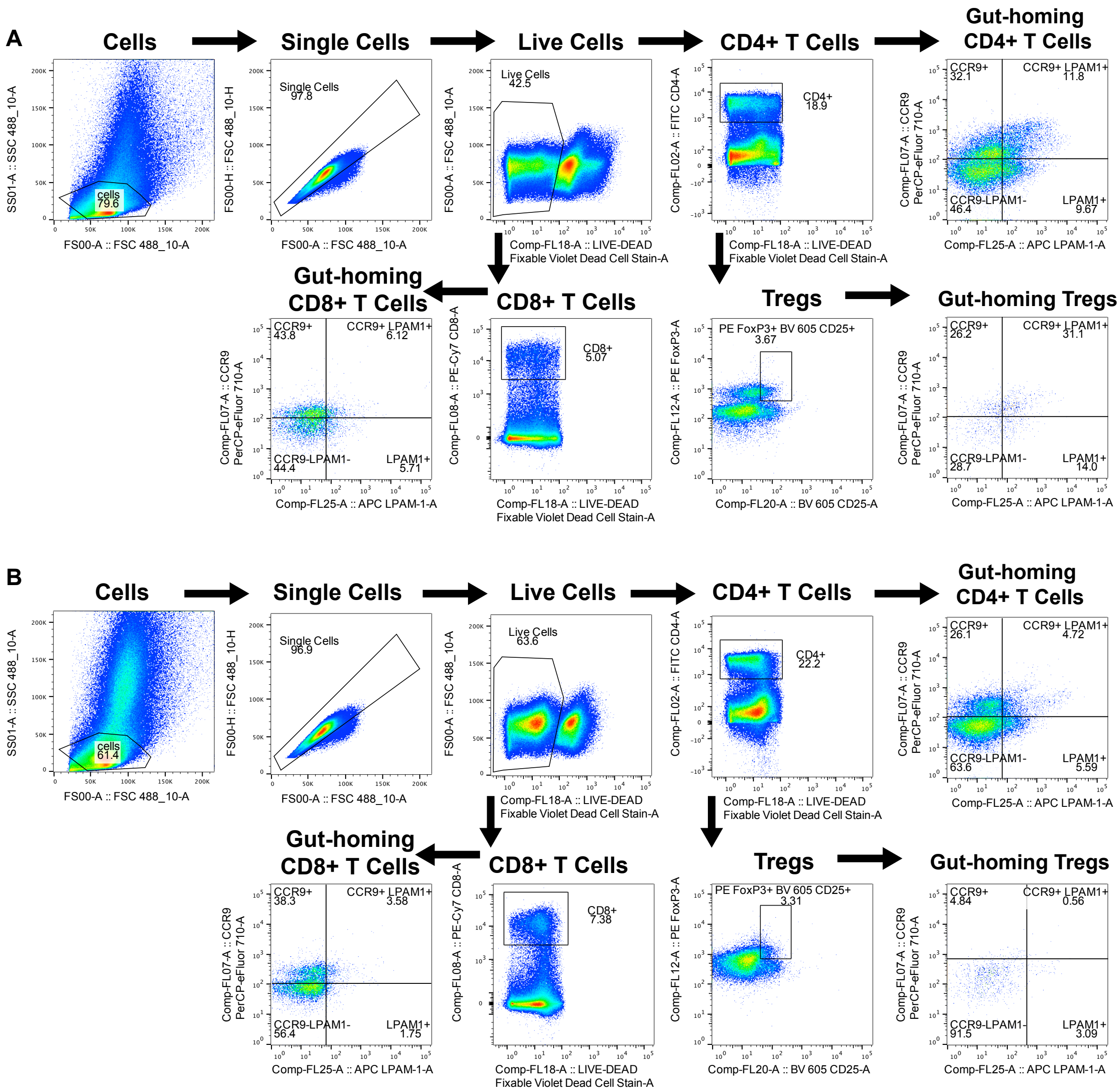

Supplemental Figure 8. *Gating scheme for Treg flow cytometry.* Cells were gated using forward scatter area and side scatter area. Singlets were identified through a forward scatter area by forward scatter height gate. Then, live cells were identified as DAPI negative using a forward scatter area by live-dead fixable violet dead cell stain area. Of live cells, CD4+ T cells were identified using a gate in dead cell stain area by FITC area. Gut-homing CD4+ T cells were identified using an APC area by PerCP-Fluor710 area gate. CD8+ T cells were identified using a gate in dead cell stain area by PE-Cy7 area gate. Gut-homing CD8+ T cells were identified using an APC area by PerCP-Fluor710 area gate. Regulatory T cells (Tregs) were identified by gating from CD4+ T cells and gating for CD4+ CD25+ FoxP3+ using BV 605 area by PE area gate. Gut-homing Tregs cells were identified using an APC area by PerCP-Fluor710 area gate **(A)**. Some samples had a shifted PE negative population and the PE FoxP3+ BV605 CD25+ gate to identify Tregs from CD4+ T cells was shifted upward as shown **(B)**.

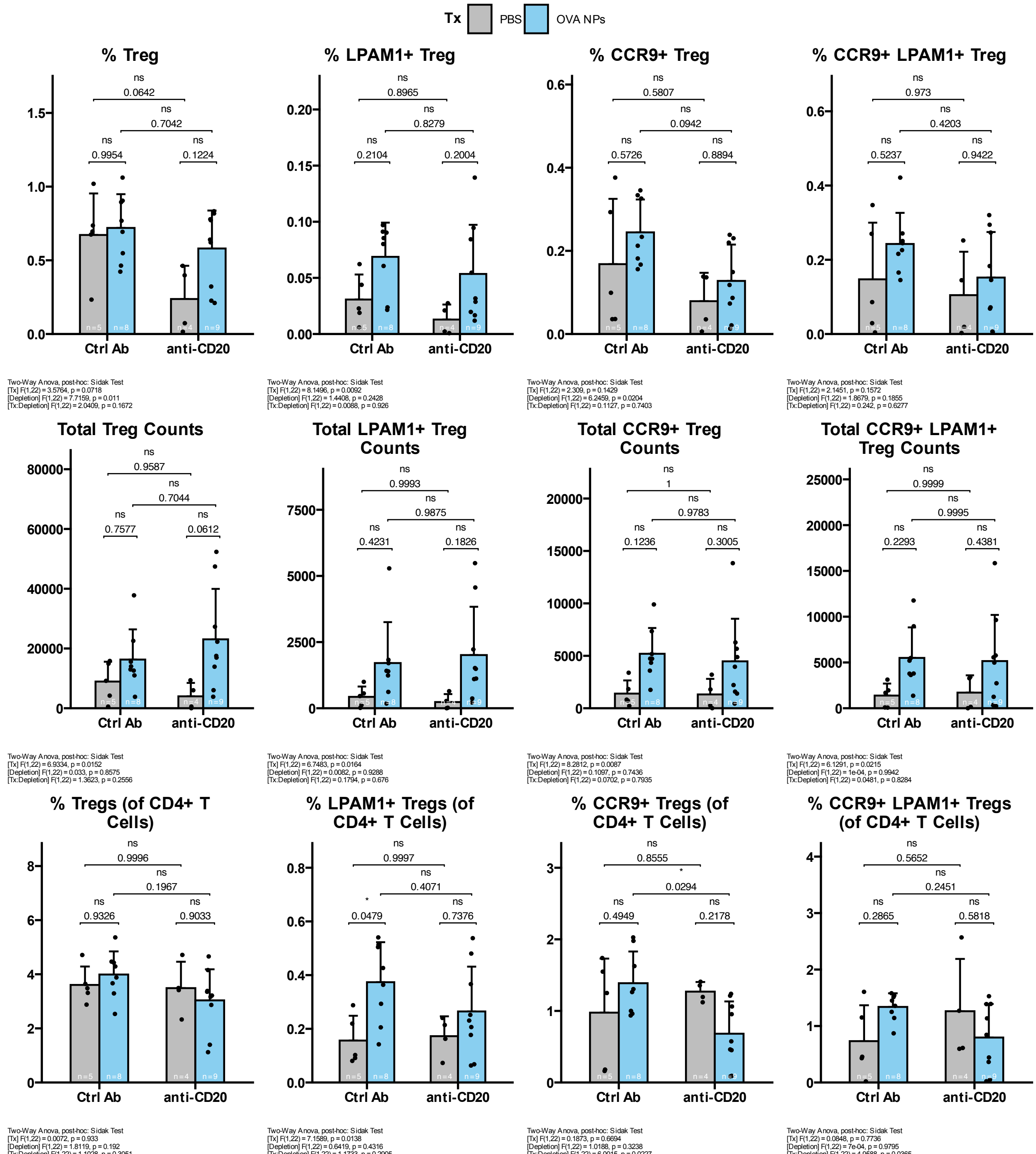

Supplemental Figure 9. *Treg flow cytometry after aeNP treatment with B cell depletion.* Following the study timeline in Figure 2A, one day after OFC 7, SILP were harvested for flow cytometry to characterize CD4+CD25+FoxP3+ Treg cells for gut-homing marker expression CCR9 and LPAM1. Error bars indicate mean ± SD; statistically significant differences were identified using a two-way ANOVA with a post-hoc Sidak Test with p-values printed above significance brackets and sample size printed at the bottom of each bar (n = 4-9).

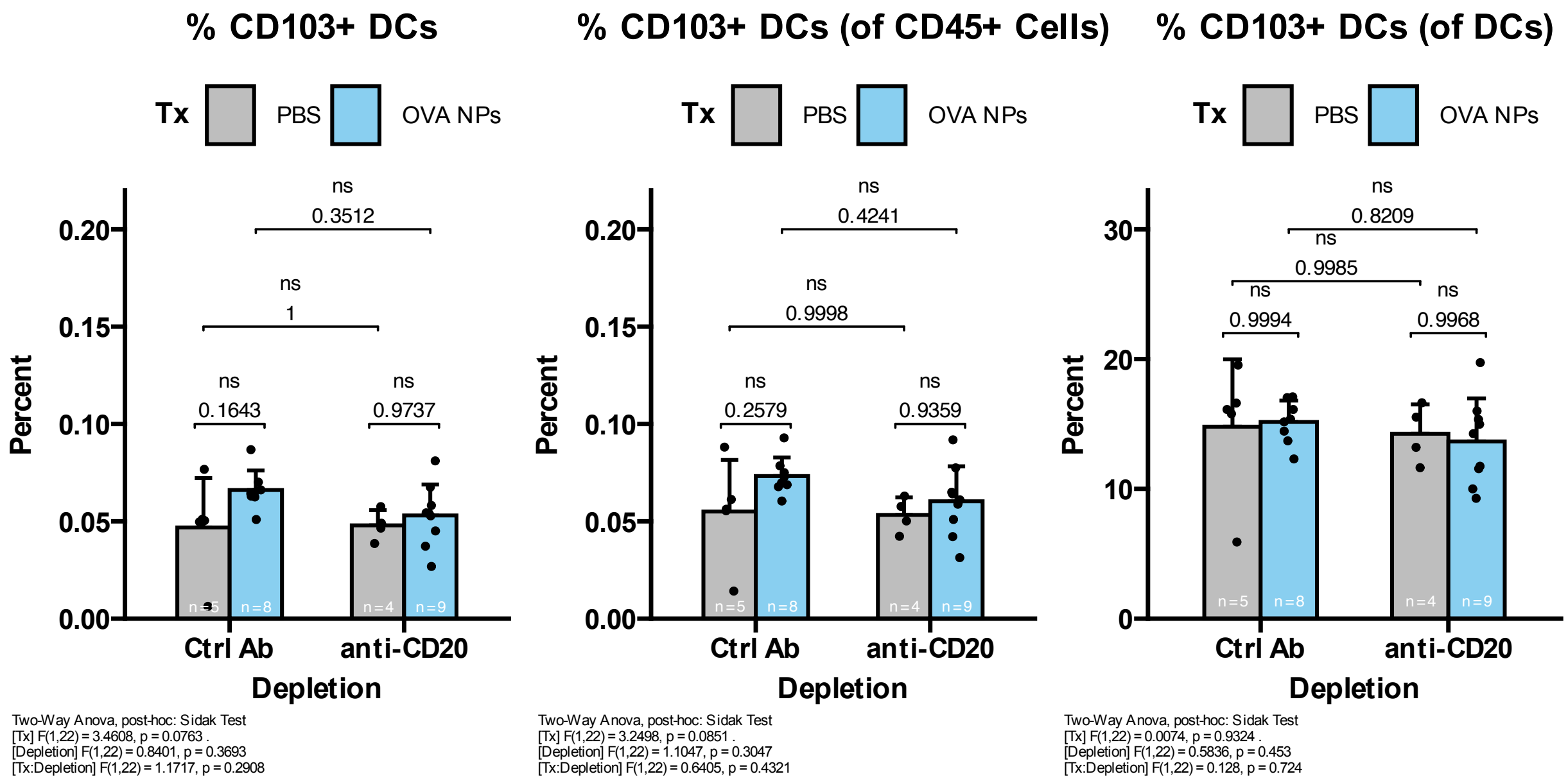

Supplemental Figure 10. *Dendritic cell flow cytometry after aeNP treatment with B cell depletion.* Following the study timeline in Figure 2A**,** one day after OFC 7, spleens were harvested for flow cytometry to characterize CD11c+ DCs for CD103 expression. Error bars indicate mean ± SD; statistically significant differences were identified using a two-way ANOVA with a post-hoc Sidak Test with p-values printed above significance brackets and sample size printed at the bottom of each bar (n = 4-9).

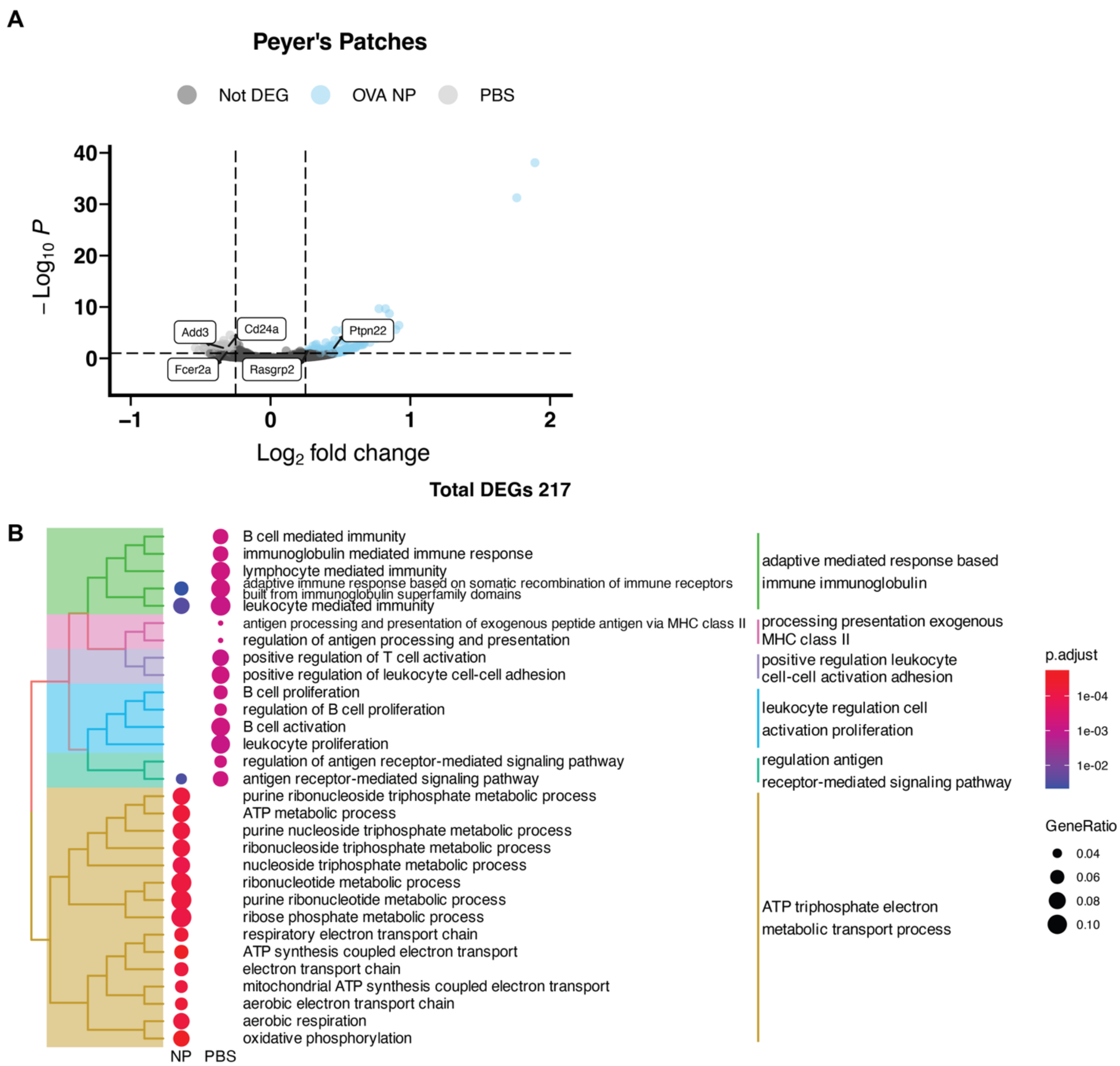

Supplemental Figure 11. *Changes in Peyer's Patches B cells after OVA NP-treatment.* Mice sensitized with OVA-alum on days 0 and 14 were treated with OVA NPs on days 28 and 42. On day 44, spleens were processed into single-cell suspensions, and B cells were isolated by magnetic-activated cell sorting of negatively-selected CD19+ cells for bulk RNA sequencing. Differentially expressed regulatory Breg markers between OVA NP- and PBS-treated mice are displayed are displayed in a volcano plot (**A**). Over Representation Analysis (ORA) of gene ontology (GO) terms in PBS and OVA NP conditions (**B**).

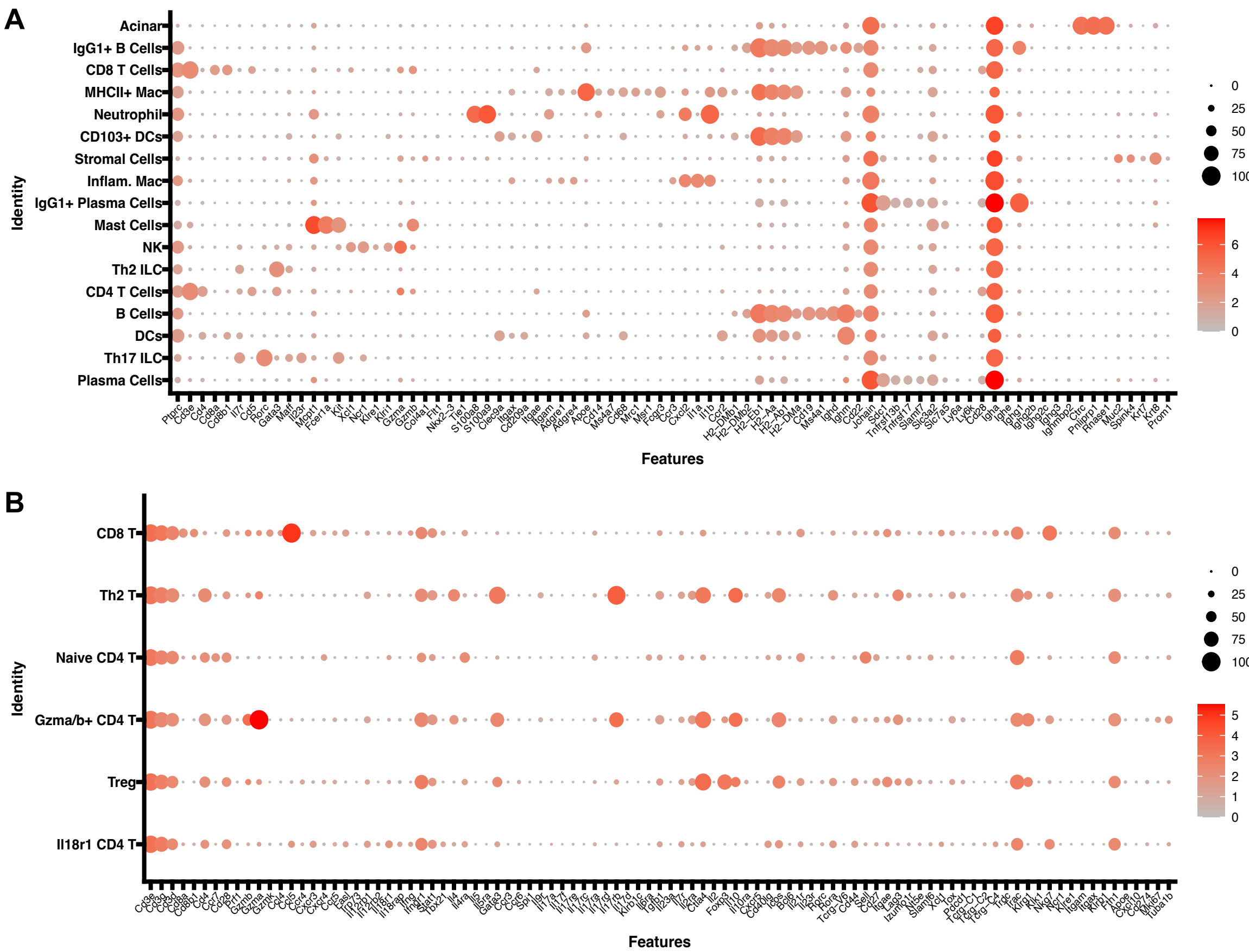

Supplemental Figure 12. Dotplot of gene markers used to identify cell clusters of all cell types **(A)** and T cell subtypes **(B)** in single-cell RNA sequencing data of SILP. The percentage of cells within each cluster identity expressing a specific gene is represented by the size of the dot, and the color of the dot is the average expression across all cells within that cluster identity.

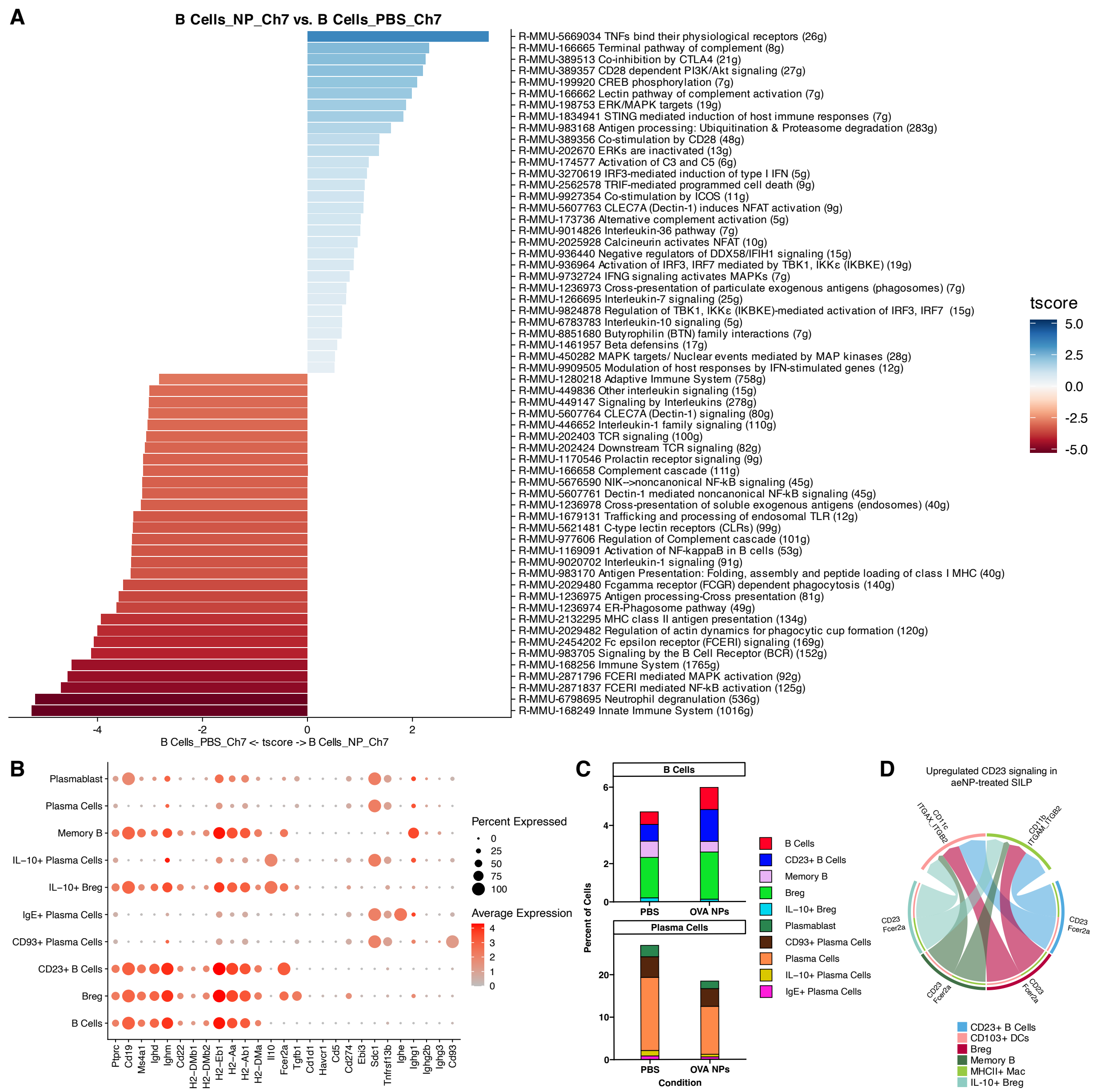

Supplemental Figure 13. *Breg subset identified in single-cell RNA sequencing data of OVA NP-treated SILP after OFC 7.* Gene set enrichment analysis comparing B cells from OVA NP-treated and allergic control (PBS) SILPs after OFC 7 using SeuratExtend **(A)**. Dotplot of gene markers used to identify B cell subsets **(B)**. Proportion of B cell subsets of all cell types in allergic controls and OVA NP-treated mice after OFC 7 **(C)**. Chord diagram of upregulated CD23 signaling in B cell subsets using receptor-ligand analysis (CellChat) in OVA NP-treated SILP compared to allergic controls at OFC 7 **(D)**.

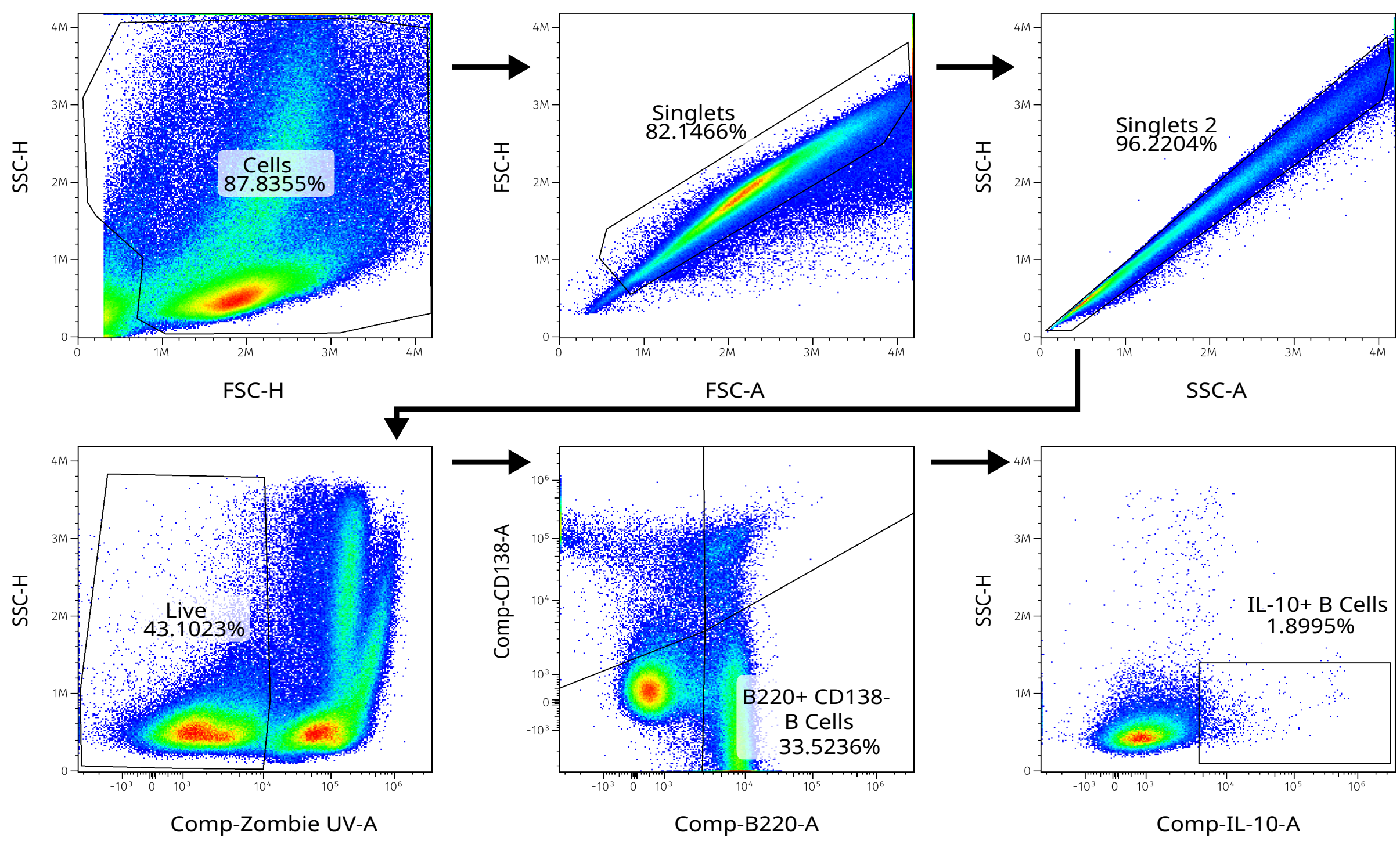

Supplemental Figure 14. *Gating scheme for IL-10 B Cell flow cytometry.* Cells were gated using forward scatter height and side scatter height. Singlets were identified through two gates: a forward scatter area by forward scatter height gate followed by a side scatter area by side scatter width gate. Then, live cells were identified as Zombie UV negative by SCC-H and Zombie UV area. Of live cells, B cells were identified as B220+CD138- using a gate in B220 area by CD138 area. IL-10+ B cells were identified by SCC-H and IL-10 area.
